# DuoMod-Net: Logarithmic balancing and geometric refinement for imbalanced semi-supervised medical image segmentation

**DOI:** 10.1016/j.patter.2026.101570

**Published:** 2026-05-27

**Authors:** Wang Bo, Along He, Ting Xue, Yue Zhang, Yi Xiao, Shiyuan Liu, Shaohua Kevin Zhou

**Affiliations:** 1 School of Biomedical Engineering, Division of Life Sciences and Medicine, University of Science and Technology of China, Hefei, Anhui, P.R. China; 2 Suzhou Institute for Advanced Research, University of Science and Technology of China, Suzhou, Jiangsu, P.R. China; 3 National Engineering Laboratory for Big Data System Computing Technology, Shenzhen University, Shenzhen, Guangdong, P.R. China; 4 Department of Radiology, Second Affiliated Hospital of Navy Medical University, Shanghai, P.R. China; 5 National Key Laboratory of Intelligent Collaborative Computing, University of Electronic Science and Technology of China, Chengdu, Sichuan, P.R. China; 6 State Key Laboratory of Precision and Intelligent Chemistry, University of Science and Technology of China, Hefei, Anhui, P.R. China; 7 Jiangsu Provincial Key Laboratory of Multimodal Digital Twin Technology, Suzhou, Jiangsu, P.R. China

**Keywords:** semi-supervised learning, class imbalance, medical image segmentation, long-tailed distribution, abdominal multi-organ segmentation, deep learning, geometric regularization

## Abstract

Class imbalance in semi-supervised medical image segmentation poses a dual challenge: it not only compromises feature learning for tail classes but also introduces significant bias in loss gradients toward the predominant background class. To address these challenges, we introduce duo-component modulation network (DuoMod-Net), a synergistic learning framework integrating two specialized components. The first component, relative logarithmic modulation (RLM), addresses the dominant gradient bias by decoupling the background magnitude from foreground balancing. It establishes the background as a neutral pivot and then applies a relative, logarithmic scaling anchored by robust percentiles to preserve the dynamic range among the foreground organs. Concurrently, the second component, disagreement-driven adaptive feature refinement (DAFR), functions as a geometric regularization mechanism. It leverages intrinsic inter-model disagreement to selectively expand the feature space during training, forcing the decision boundary to recede. This expansion is removed at inference, establishing a safety margin that enhances detection reliability. Extensive validation across varying data regimes (5%, 10%, and 20%) demonstrates that DuoMod-Net yields substantial improvements on tail classes, increases detection reliability by minimizing catastrophic failures, and maintains robust zero-shot generalization on unseen datasets.

## Highlights

• DuoMod-Net tackles class imbalance in semi-supervised medical image segmentation
• Logarithmic modulation balances learning by decoupling arbitrary background noise
• Disagreement-driven feature refinement creates a geometric safety margin
• DuoMod-Net greatly improves detection reliability for rare tail-class organs

## The bigger picture

Artificial intelligence (AI) has the potential to revolutionize medical image analysis, but its success relies heavily on massive amounts of manually annotated data. To reduce this annotation burden, researchers use semi-supervised learning to train models on readily available unlabeled data. However, real-world clinical data are overwhelmingly imbalanced: large organs and background regions dominate the scans, while crucial small or rare anatomical structures (the tail classes) are scarcely represented. Standard AI models tend to be overwhelmed by the frequent classes, often leading to severe detection failures for rare but vital organs.

To overcome this dual challenge of biased learning and sparse features, we developed duo-component modulation network (DuoMod-Net). Instead of simply forcing the AI to pay more attention to rare organs—which often amplifies noise—our framework structurally rethinks the learning process. First, it decouples arbitrary background noise, allowing the model to balance its learning focus exclusively among the actual anatomical structures. Second, it monitors when the AI is uncertain about its predictions and uses this disagreement to intentionally push the boundaries of common organs outward during training. During clinical inference, this expansion is retracted, leaving a protective safety margin that minimizes the risk of small organs being misclassified. By creating this synergistic environment, our method substantially improves the reliable detection of rare anatomical structures. Beyond medical imaging, the core principles of decoupling background noise and geometrically enforcing safety margins offer a robust blueprint for tackling severe class imbalance in broader data science applications.

## Graphical abstract

**Figure:**
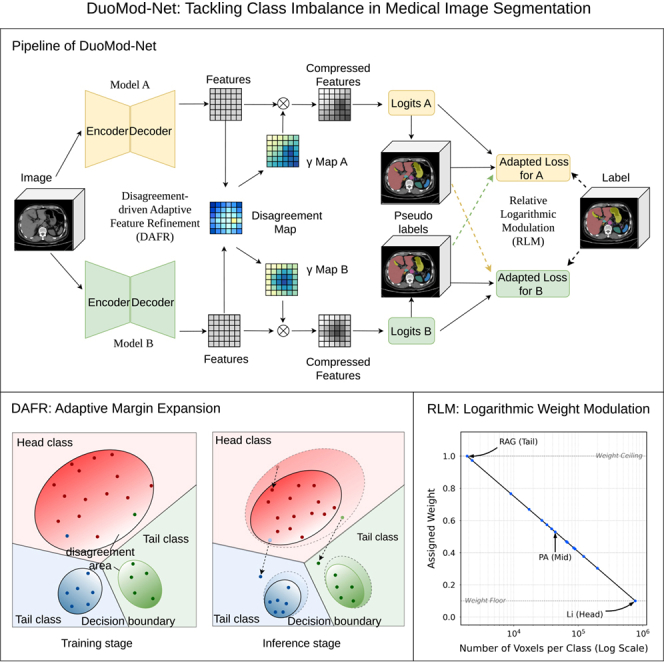

## Teaser

Training AI on medical images is challenging because rare organs are often overlooked by standard models. Bo et al. introduce DuoMod-Net to address this severe class imbalance. By decoupling background noise to stabilize learning gradients and dynamically expanding feature spaces to create a geometric safety margin for rare structures, this framework improves the reliable detection of underrepresented organs, advancing robust and comprehensive medical image analysis under limited data conditions.

## Introduction

**Figure 1:**
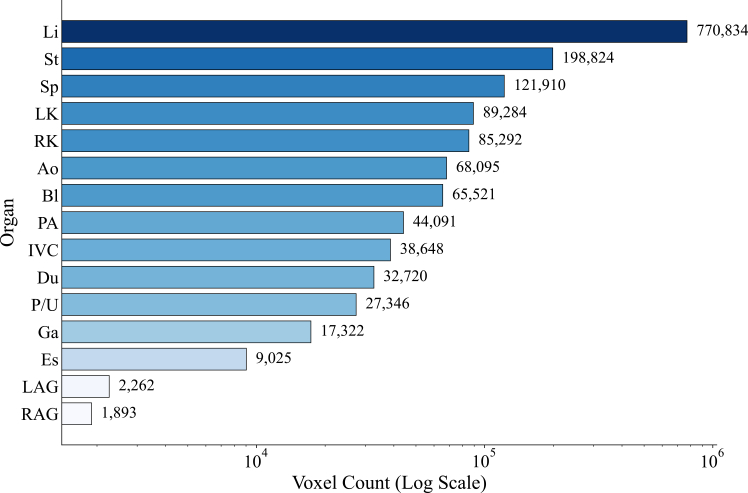
Voxel distribution of the 15 foreground classes in the AMOS dataset The voxel counts for each class are plotted on a logarithmic scale to explicitly illustrate the severe class imbalance across the 15 foreground categories.

Medical image segmentation^1^ faces a fundamental challenge: the inherent scarcity of high-quality pixel-wise annotations, which are both labor-intensive to create and require specialized clinical expertise. Semi-supervised learning (SSL) has consequently gained prominence as a promising solution, exploiting readily available unlabeled data to reduce dependence on scarce annotated samples.^2^^,^^3^^,^^4^^,^^5^^,^^6^^,^^7^^,^^8^ However, existing SSL methodologies predominantly rely on the assumption of balanced class distributions—a condition rarely satisfied in clinical practice, especially in applications such as abdominal organ segmentation,^9^^,^^10^^,^^11^ where long-tailed distributions are common (see Figure 1). Such an imbalance presents a dual challenge for model training. First, it induces biased loss gradients dominated by the head classes and the background. Second, it results in sparse and suboptimal feature representations for the under-represented tail classes (quantitatively defined via voxel frequency rankings and cross-dataset intersection to maintain evaluation consistency; see supplemental methods for the specific selection protocol and class lists).

Existing approaches attempt to address these challenges^12^ but face notable limitations. To address the biased loss gradients—a problem arising from the loss function being numerically dominated by frequent classes—various approaches employ re-balancing strategies to modulate each class’s contribution to the overall gradient. These strategies fall into two broad categories. The first, based on statistical frequencies (either static^13^^,^^14^ or dynamic^15^), often exhibits a common limitation: it treats the background class, whose voxel count is highly variable and dependent on the cropping window rather than anatomy, as a valid input for its calculations. By anchoring their weights to this context-dependent maximum value (i.e., the background),^15^ these methods can render the loss sensitive to noise and restrict the dynamic range available for the actual foreground classes. The second category employs dynamic heuristics, re-weighting based on complex performance indicators such as model confidence^16^ or gradient analysis,^17^ which can be unstable or less consistent in low-data regimes. Concurrently, to address the sparse feature representations, separate efforts have also fallen into two categories. Some approaches are static, such as fixed refinement modules,^18^ which provide limited adaptation. Alternative approaches introduce heavyweight extrinsic mechanisms, such as dedicated prototype classifiers^19^ or entirely separate data-flow pipelines and auxiliary heads.^20^ However, these feature-level strategies, particularly within dual-model SSL frameworks (e.g., cross pseudo supervision [CPS]^6^), largely underexploit the rich, intrinsic interaction signal (i.e., inter-model disagreement) that is already present and provides a useful proxy for uncertainty.

To address this dual challenge in a synergistic manner, we developed duo-component modulation network (DuoMod-Net), a framework that integrates two specialized components. To mitigate the biased loss gradients, we propose a solution that avoids the limitations of existing loss strategies. We introduce the relative logarithmic modulation (RLM) module. RLM is founded on two core principles: first, foreground-background decoupling to mitigate background noise interference, and second, a relative, logarithmic framework to stably address the remaining foreground imbalance. RLM calculates weights based on the foreground classes’ statistics, establishing the background as a neutral pivot rather than a dominant factor, and then applies a logarithmic scaling relative to a robust range anchored by statistical percentiles. This dual mechanism effectively mitigates background noise while preserving a high-resolution dynamic range for the tail classes. To address feature sparsity, we introduce the disagreement-driven adaptive feature refinement (DAFR) module. Functionally operating as an uncertainty-guided feature perturbation mechanism, DAFR employs a train-test inversion protocol to leverage intrinsic inter-model disagreement. By selectively amplifying ambiguous features during training, it drives the model to learn a robust boundary in an expanded space. Notably, this amplification primarily targets samples from dominant (head) classes. We deliberately attenuate the amplification intensity for tail classes, as their inherently high feature variance renders them susceptible to noise expansion, potentially destabilizing the decision boundary. At inference, this amplification is removed, establishing a geometric safety margin that enhances detection reliability for under-represented classes. Unlike approaches that treat class imbalance and feature ambiguity in isolation, our core contribution lies in the synergistic integration of these domains. Specifically, the stable gradient dynamics anchored by RLM provide the optimization foundation, enabling DAFR to impose directed geometric perturbations with enhanced efficacy and stability.

Our principal contributions are 3-fold.

• A synergistic framework, DuoMod-Net, that addresses both loss gradient bias and feature sparsity through two specialized components: RLM (background-decoupled logarithmic balancing) and DAFR (disagreement-guided geometric regularization).
• A standardized benchmarking protocol that addresses prior methodological inconsistencies by re-evaluating methods on a standardized, resolution-independent pipeline, ensuring consistent comparisons.
• Extensive validation across varying data regimes (5%, 10%, and 20% labeled data), demonstrating that DuoMod-Net achieves statistically significant improvements on in-domain benchmarks, exhibits reliable detection in extreme low-data settings, and maintains robust zero-shot generalization on unseen datasets.

## Results

### Experimental setup

We evaluated our proposed strategies on three public, long-tailed multi-organ segmentation benchmarks: AMOS,^9^ WORD,^10^ and FLARE 2022.^11^ The AMOS dataset (360 scans) and WORD dataset (150 scans) were utilized as the primary datasets for training and evaluation. AMOS is a diverse benchmark comprising both computed tomography (CT) and MRI scans, while WORD is a CT dataset featuring a different set of abdominal organs. For the analysis of generalization, we also performed zero-shot, cross-dataset evaluation on the FLARE 2022 validation set (50 scans). A detailed breakdown of the organ classes for each dataset is provided in the supplemental methods. To assess performance under limited supervision, we primarily evaluated our method using a 5% labeled setting and further validated its scalability across 10% and 20% label proportions.

To evaluate the performance of our framework, DuoMod-Net, we benchmarked it against a selection of representative competing methods. These methods are organized into two distinct categories. The first category includes general-purpose SSL methods that do not explicitly address class imbalance, such as UA-MT,^3^ DST,^21^ DMD,^22^ SLCNet,^23^ DyCON,^24^ and the CPS^6^ baseline. The second group includes recent class-imbalanced SSL (CISSL) methods specifically designed for this challenge, such as Adsh,^25^ CLD,^13^ DHC,^15^ feature clusters compression (FCC),^18^ GA-loss^17^ and SKCDF.^20^ To facilitate a standardized comparison, we re-implemented the competing methods and trained them within our unified pipeline. This approach standardizes the data preprocessing and training protocols and specifies that metrics are calculated by resampling segmentations back to their original physical resolution.

We quantitatively evaluate segmentation performance using two types of standard metrics: the Dice similarity coefficient (DSC) and the average surface distance (ASD). Notably, the conventional calculation of ASD (used in our main tables) is typically conditional on successful detection, ignoring instances in which the organ is entirely missed. To explicitly penalize such detection failures—which are frequent in tail classes—we additionally adopt the penalized ASD protocol (denoted as ASD^pen^), as widely implemented in major benchmarks such as MSD,^26^ for detailed failure analysis. This metric assigns a maximum spatial distance penalty to non-detected organs, thereby providing a more holistic assessment of segmentation reliability. To provide a stratified analysis, we report these metrics averaged across the foreground classes (Dice and ASD) and also averaged exclusively over a predefined set of challenging tail classes (tail-class Dice [Dice_tail_] and ASD_tail_), which are explicitly defined in the supplemental methods. Furthermore, to facilitate a clinically meaningful and consistent comparison, the surface distance metrics (ASD, ASD_tail_, and ASD^pen^) are computed in the physical space (in millimeters). This approach avoids the ambiguous and inconsistent results of voxel-based distances, which are not comparable across scans with different voxel spacings. To assess the statistical significance of performance improvements, we perform a paired Wilcoxon signed-rank test between our method and each competitor. To account for multiple comparisons, *p* values are subsequently adjusted using the Benjamini-Hochberg procedure, with an adjusted *p* < 0.05 considered statistically significant.

### Comparison with competing methods

We first evaluated the in-domain performance of our framework, DuoMod-Net, against the competing methods on the AMOS and WORD datasets, with detailed results presented in Table 1. To contextualize the performance gap caused by label scarcity, we also include the results of a fully supervised V-Net trained on 100% labeled data as an upper-bound reference (first row). Detailed per-class results for these datasets, as well as for the cross-dataset evaluation on FLARE22, are provided in Tables S1–S6.

**Table 1:** Quantitative comparison of Dice (%) and ASD (mm) on the AMOS and WORD datasets under the 5% labeled setting

Category	Method	AMOS (5% labeled)				WORD (5% labeled)			
		Dice	ASD	Dice_tail_	ASD_tail_	Dice	ASD	Dice_tail_	ASD_tail_
V-Net (100%)	–	83.37_0.22_	0.73_0.04_	71.90_0.05_	1.06_0.04_	84.19_0.10_	0.89_0.08_	71.75_0.08_	1.40_0.11_
General	UA-MT^3^	$54.45_{1.06}^{***}$	$8.87_{0.92}^{***}$	$39.20_{1.71}^{***}$	$9.20_{0.99}^{***}$	$47.13_{1.46}^{***}$	$14.08_{1.45}^{***}$	$23.20_{0.94}^{***}$	$14.19_{3.44}^{***}$
	CPS^6^	$57.98_{1.28}^{***}$	$5.52_{0.56}^{***}$	$41.16_{0.40}^{***}$	$5.24_{0.65}^{***}$	$71.28_{0.15}^{***}$	$3.91_{0.24}^{*}$	$54.59_{0.54}^{***}$	3.76_0.22_
	DST^21^	$45.97_{0.66}^{***}$	$11.41_{3.04}^{***}$	$26.83_{3.10}^{***}$	$17.53_{12.02}^{***}$	$66.19_{1.56}^{***}$	$7.62_{3.45}^{***}$	$47.18_{3.77}^{***}$	$11.72_{9.84}^{***}$
	DMD^22^	$60.69_{0.71}^{***}$	$4.93_{0.18}^{***}$	$45.26_{0.65}^{***}$	$4.29_{0.21}^{***}$	$71.22_{0.42}^{***}$	$3.93_{0.22}^{*}$	$54.32_{0.48}^{***}$	3.81_0.36_
	SLCNet^23^	$58.53_{0.85}^{***}$	$6.18_{1.27}^{***}$	$43.76_{0.65}^{***}$	$6.82_{1.48}^{***}$	$70.47_{1.21}^{**}$	$4.77_{0.46}^{**}$	$51.91_{2.06}^{***}$	$4.07_{0.42}^{*}$
	DyCON^24^	$48.05_{1.56}^{***}$	$9.10_{1.68}^{***}$	$26.39_{0.69}^{***}$	$10.79_{2.69}^{***}$	$31.78_{4.77}^{***}$	$30.59_{3.61}^{***}$	$5.30_{3.52}^{***}$	$31.67_{3.18}^{***}$
Imbalanced	Adsh^25^	$57.97_{1.44}^{***}$	$5.54_{0.29}^{***}$	$39.67_{2.33}^{***}$	$6.01_{0.23}^{***}$	$71.18_{0.64}^{***}$	$4.16_{0.77}^{**}$	$54.27_{0.60}^{***}$	$4.04_{0.23}^{**}$
	CLD^13^	$61.33_{0.57}^{***}$	$4.77_{0.37}^{***}$	$44.09_{1.05}^{***}$	$4.69_{0.21}^{***}$	$71.84_{0.42}^{***}$	$3.76_{0.26}^{**}$	$54.92_{0.29}$	$3.90_{0.11}^{*}$
	DHC^15^	$58.52_{0.45}^{***}$	$7.11_{0.54}^{***}$	$45.31_{1.52}^{***}$	$5.16_{0.35}^{***}$	$61.53_{7.10}^{***}$	$11.65_{4.86}^{***}$	$46.34_{6.67}^{***}$	$6.10_{1.40}^{***}$
	FCC^18^	$59.53_{1.21}^{***}$	$4.70_{0.30}^{***}$	$40.90_{0.98}^{***}$	$4.47_{0.10}^{***}$	$71.58_{0.53}^{***}$	$3.55_{0.65}^{*}$	$55.16_{0.79}^{***}$	3.61_0.15_
	GA-loss^17^	$62.10_{2.25}^{***}$	$4.98_{0.18}^{***}$	$46.43_{3.29}^{***}$	$4.74_{0.53}^{***}$	$70.75_{0.20}^{***}$	$4.37_{0.10}^{***}$	$54.27_{0.42}^{***}$	$3.83_{0.03}^{*}$
	SKCDF^20^	$57.16_{0.83}^{***}$	$7.80_{0.80}^{***}$	$41.38_{1.52}^{***}$	$7.83_{0.75}^{***}$	$70.92_{0.64}^{***}$	$5.94_{0.59}^{***}$	$54.26_{1.26}^{***}$	$5.57_{0.28}^{***}$
	ours	65.22_0.59_	4.40_0.13_	51.37_0.57_	3.39_0.37_	72.66_0.25_	3.76_0.72_	56.37_0.37_	3.67_0.15_

Results are presented as mean_SD_ over three independent runs. Asterisks denote statistical significance from a paired Wilcoxon signed-rank test against our method (∗p < 0.05, ∗∗p < 0.01, and ∗∗∗p < 0.001) after Benjamini-Hochberg correction. V-Net (100%) serves as the fully supervised performance upper bound.

On the AMOS 5% labeled dataset, DuoMod-Net demonstrates notable improvements. As shown in Table 1, our framework achieves leading results across the four evaluated metrics (Dice, ASD, Dice_tail_, and ASD_tail_). These improvements are statistically significant, with *p* values (indicated by ∗∗∗ in the competitor rows) indicating a significant improvement over the other methods (*p* < 0.001). The most pronounced gains are observed in the challenging tail-class metrics. Our model achieves a Dice_tail_ of 51.37%, an absolute improvement of 4.94% over the closest competitor, GA-loss (46.43%). Similarly, our ASD_tail_ of 3.39 mm is significantly lower than that of other approaches.

On the WORD 5% labeled dataset, the method continues to show competitive performance, achieving leading results in 3 out of 4 metrics, including a statistically significant advantage in Dice (72.66%) and Dice_tail_ (56.37%). The results for surface distance metrics on this dataset are more nuanced. For ASD, while our method (3.76 mm) yields a slightly higher mean than FCC (3.55 mm), a detailed breakdown (Table S5) reveals that this numerical increase is driven primarily by specific outliers in the left kidney class (see the geometric analysis and boundary behavior section for a detailed discussion of this phenomenon). Despite the skewing influence of these outliers on the mean, a paired statistical test (*p* < 0.05) demonstrates a statistically significant advantage for our method, indicating greater consistency on a per-sample basis. For the ASD_tail_ metric, we note that the result from FCC (3.61 mm) is numerically lower than ours (3.67 mm). This difference, however, was found to be not statistically significant (*p* = 0.1103). While other methods may offer comparable performance on specific surface distance metrics, our framework consistently delivers statistically significant and robust performance in the core metrics of volumetric overlap (Dice).

Overall, our method achieves the leading performance in 7 out of 8 in-domain metric categories, demonstrating its robustness and effectiveness, particularly in enhancing volumetric segmentation of tail classes.

### Robustness across varying label regimes

To verify the generalization capability of DuoMod-Net beyond the 5% labeled low-data regime, we extended our evaluation to scenarios with 10% and 20% labeled data. These experiments assess whether our core contributions, the RLM and DAFR modules, retain their effectiveness when supervision becomes more abundant and imply whether the method suffers from premature performance saturation. We leverage the fully supervised upper bound (100% labeled data) to quantify the extent to which our method bridges the supervision gap. Table 2 summarizes the quantitative results on the AMOS dataset, while detailed comparisons for the WORD dataset exhibit consistent trends, as shown in Table S7.

**Table 2:** Experimental results on the AMOS dataset under the 10% and 20% labeled data settings

Category	Method	AMOS 10% labeled				AMOS 20% labeled			
		Dice	ASD	Dice_tail_	ASD_tail_	Dice	ASD	Dice_tail_	ASD_tail_
V-Net (100%)	–	83.37_0.22_	0.73_0.04_	71.90_0.05_	1.06_0.04_	83.37_0.22_	0.73_0.04_	71.90_0.05_	1.06_0.04_
General	UA-MT^3^	$66.60_{1.92}^{***}$	$7.84_{1.99}^{***}$	$52.63_{1.36}^{***}$	$7.37_{2.58}^{***}$	$67.66_{3.39}^{***}$	$5.20_{1.07}^{***}$	$52.04_{4.14}^{***}$	$5.01_{0.80}^{***}$
	CPS^6^	$74.56_{0.36}^{***}$	$2.51_{0.24}^{*}$	$58.51_{0.31}^{***}$	$2.33_{0.09}^{***}$	$76.76_{0.27}^{***}$	$1.58_{0.11}^{***}$	$62.40_{0.17}^{***}$	$2.16_{0.16}^{***}$
	DST^21^	$69.25_{0.42}^{***}$	$4.74_{1.51}^{***}$	$50.18_{2.15}^{***}$	$7.49_{6.15}^{***}$	$68.73_{1.97}^{***}$	$3.17_{0.49}^{***}$	$50.45_{2.18}^{***}$	$3.60_{0.40}^{***}$
	DMD^22^	$73.94_{1.04}^{***}$	$2.35_{0.29}^{***}$	$58.53_{1.39}^{***}$	$2.42_{0.21}^{***}$	$76.43_{0.01}^{***}$	$1.70_{0.04}^{***}$	$62.03_{0.11}^{***}$	$2.09_{0.07}^{***}$
	SLCNet^23^	$73.98_{0.56}^{***}$	$2.55_{0.51}^{***}$	$59.72_{0.18}^{***}$	$2.47_{0.42}^{***}$	$74.91_{1.89}^{***}$	$1.67_{0.13}^{***}$	$62.64_{0.18}^{***}$	$2.05_{0.12}^{***}$
	DyCON^24^	$61.97_{1.36}^{***}$	$4.76_{1.45}^{***}$	$44.17_{2.74}^{***}$	$7.26_{2.83}^{***}$	$54.36_{3.50}^{***}$	$5.00_{0.19}^{***}$	$24.40_{6.41}^{***}$	$8.74_{1.46}^{***}$
Imbalanced	Adsh^25^	$73.72_{0.11}^{***}$	$2.29_{0.19}^{***}$	$58.14_{0.66}^{***}$	$2.42_{0.13}^{***}$	$75.92_{0.46}^{***}$	$1.83_{0.11}^{***}$	$61.50_{0.70}^{***}$	$2.21_{0.13}^{***}$
	CLD^13^	$75.10_{0.30}^{***}$	$2.42_{0.11}^{*}$	$59.16_{0.60}^{***}$	$2.30_{0.05}^{***}$	$76.86_{0.14}^{***}$	$1.54_{0.05}^{**}$	$62.62_{0.33}^{***}$	$2.06_{0.02}^{***}$
	DHC^15^	$70.94_{0.73}^{***}$	$5.78_{1.39}^{***}$	$60.60_{1.35}^{***}$	$3.25_{0.58}^{***}$	$73.79_{1.00}^{***}$	$3.45_{0.43}^{***}$	$63.50_{0.87}^{***}$	$2.58_{0.31}^{***}$
	FCC^18^	$74.83_{0.33}^{***}$	$2.31_{0.10}^{*}$	$59.32_{0.36}^{***}$	$2.23_{0.03}^{***}$	$73.73_{0.53}^{***}$	$1.97_{0.12}^{***}$	$60.19_{1.13}^{***}$	$2.28_{0.03}^{***}$
	GA-loss^17^	$75.97_{0.70}^{***}$	$2.13_{0.25}^{*}$	$60.33_{0.99}^{***}$	$2.06_{0.09}^{***}$	$75.99_{0.34}^{***}$	$2.10_{0.17}^{***}$	$61.87_{0.47}^{***}$	$2.05_{0.07}^{***}$
	SKCDF^20^	$73.18_{0.21}^{***}$	$3.25_{0.19}^{***}$	$57.74_{0.51}^{***}$	$3.15_{0.37}^{***}$	$73.77_{0.31}^{***}$	$2.95_{0.35}^{***}$	$59.89_{0.38}^{***}$	$3.71_{0.47}^{***}$
	ours	76.81_0.07_	2.16_0.08_	62.74_0.48_	1.94_0.07_	78.20_0.12_	1.47_0.07_	65.12_0.35_	1.83_0.15_

Performance is evaluated using Dice (%) and ASD (mm). Results are presented as mean_SD_ over three independent runs. Asterisks denote statistical significance from a paired Wilcoxon signed-rank test against our method (∗p < 0.05, ∗∗p < 0.01, and ∗∗∗p < 0.001) after Benjamini-Hochberg correction. V-Net (100%) serves as the fully supervised performance upper bound.

Quantitative analysis reveals that while increasing label availability generally benefits model performance, several competing methods (e.g., DST and FCC) exhibit performance plateaus or slight fluctuations between the 10% and 20% settings. In contrast, DuoMod-Net maintains a consistent performance advantage across both regimes. In the 20% setting on AMOS, our method achieves a Dice of 78.20% and a Dice_tail_ of 65.12%, surpassing the closest method, DHC (63.50%), and the standard baseline, CPS (62.40%). This consistent trend is mirrored in the WORD dataset with the 10% setting, where our method outpaces the most competitive specialized approach, CLD (e.g., 62.82% vs. 61.24% in Dice_tail_). This indicates that even in higher-data regimes, the class imbalance issue remains a critical bottleneck, and our modulation strategies effectively mitigate this to extract more discriminative features for under-represented classes.

Comparing our results with the fully supervised upper bound (V-Net using 100% labels) further reveals the data efficiency of our approach. With only 20% of the annotations, DuoMod-Net recovers a substantial portion of the fully supervised performance—reaching 93.8% (78.20% vs. 83.37%) of the upper bound’s Dice on AMOS and 96.2% (81.00% vs. 84.19%) on WORD. Notably, this high recovery rate extends to the Dice_tail_ (90.6% on AMOS and 93.2% on WORD), markedly narrowing the supervision gap on challenging tail classes.

### Robust generalization to unseen datasets

To evaluate the generalization and robustness of our framework, DuoMod-Net, we conducted a zero-shot, cross-dataset evaluation. In this setup, the methods were trained exclusively on the AMOS 5% labeled dataset and then directly applied to the unseen FLARE22 validation set, without any retraining or fine-tuning. This test assesses the models’ ability to generalize their learned anatomical understanding to a new domain with different data characteristics.

The results, presented in Table S8, demonstrate a consistent advantage for our method. A detailed per-class breakdown for this zero-shot test is available in Tables S3 and S6. Our framework achieves the leading mean performance across the four evaluated metrics, consistently outperforming the competitors. The most notable result is observed in the tail-class performance. Our model achieves a Dice_tail_ of 43.53%, representing a 3.62% absolute improvement over the closest method, GA-loss (39.91%). Furthermore, our method also obtains the lowest surface distance scores, with an ASD of 5.36 mm and an ASD_tail_ of 5.52 mm, both of which are significantly lower than the competing approaches, as demonstrated by the paired tests in Table S8.

These results indicate that our framework, by effectively addressing the class imbalance with the DAFR and RLM modules, learns more robust and generalizable feature representations. This allows it to maintain stable performance even when faced with novel data distributions rather than simply overfitting to the source dataset.

### Analysis of failure modes and boundary reliability

**Figure 2:**
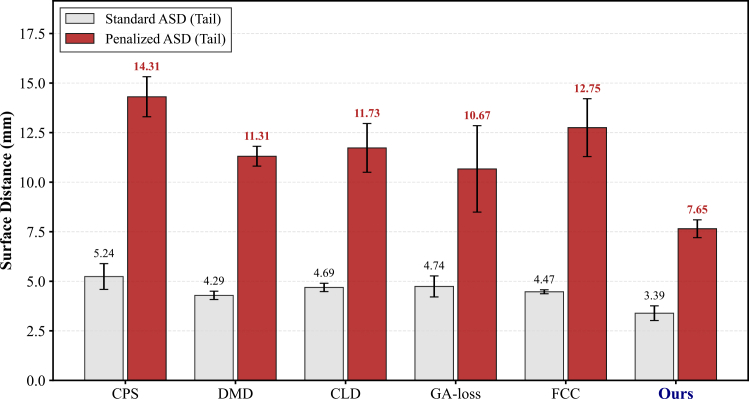
Comparison of detection reliability on AMOS tail classes under the 5% labeled setting The comparison evaluates standard ASD (gray bars) against penalized ASD (red bars). Penalized ASD incorporates a 100 mm penalty for missed organ detections to explicitly quantify model reliability. Error bars represent the standard deviation over three independent runs.

Standard surface distance metrics often rely on the assumption of successful detection, excluding failed cases (NaNs) from the calculation. While this focuses on boundary precision for detected instances, it masks severe detection failures, creating an inherent survivorship bias in the evaluation. Therefore, in this section, we explicitly report the ASD^pen^, where a maximum distance penalty of 100 mm is assigned to missed organs. This metric serves as a proxy for detection reliability. The comparative results for the tail classes on the AMOS 5% dataset are visualized in Figure 2, and the full numerical results for both AMOS and WORD datasets across the data regimes are provided in Tables S9 and S10.

On the AMOS dataset, which features severe class imbalance and mixed modalities, the advantage of DuoMod-Net is systematic. Under the most challenging 5% setting, competitors such as CLD and GA-loss exhibit sharply degraded performance (${\text{ASD}}_{\text{tail}}^{\text{pen}}>10$ mm), indicating frequent organ misses. In contrast, our method maintains a significantly lower metric (7.65 mm), suggesting that the DAFR module provides a stable safety net against model collapse. Notably, this stable trend persists as labeled data increases: at 10% and 20% settings, our framework continues to yield the lowest ${\text{ASD}}_{\text{tail}}^{\text{pen}}$ scores compared to the competitors (see Table S9).

On the WORD dataset, the results highlight a more competitive landscape. At the scarcest 5% setting, our method achieves an ${\text{ASD}}_{\text{tail}}^{\text{pen}}$ of 4.29 mm, remaining comparable to the leading competitor, FCC (3.81 mm). While FCC exhibits a slight advantage in boundary compactness in this specific low-data regime, notably, DuoMod-Net retains a higher Dice_tail_ (56.37% vs. 55.16%). This indicates that our method maintains robust object detection without sacrificing volumetric coverage. Furthermore, as supervision increases, our method demonstrates enhanced scalability. At 10% labels, DuoMod-Net’s ${\text{ASD}}_{\text{tail}}^{\text{pen}}$ is on par with the lowest baseline (2.29 vs. 2.28 mm), and at 20% labels, it achieves the leading performance (1.83 mm), suggesting that our framework effectively combines detection reliability with precise boundary refinement.

### Performance on tail classes

**Figure 3:**
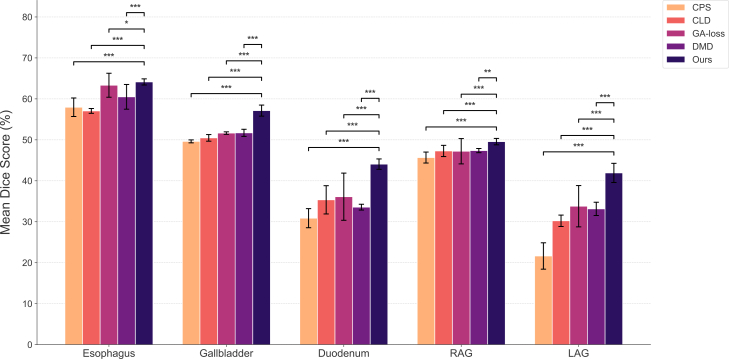
Comparison of mean Dice scores across five tail classes on the AMOS dataset (5% labeled) Quantitative performance is evaluated for the esophagus, gallbladder, duodenum, RAG, and LAG classes. Our method is benchmarked against four competitive baselines: CPS, CLD, GA-loss, and DMD. Each bar represents the mean Dice value, and error bars indicate the standard deviation over three independent runs. Asterisks denote statistical significance from a paired Wilcoxon signed-rank test against our method (∗p < 0.05, ∗∗p < 0.01, and ∗∗∗p < 0.001) after Benjamini-Hochberg correction.

To assess our model’s capability to segment challenging and rare anatomical structures, we performed a detailed analysis on the AMOS dataset under the 5% labeled data setting. This evaluation focused on five representative tail classes: esophagus, gallbladder, duodenum, right adrenal gland (RAG), and left adrenal gland (LAG). We compared our method, DuoMod-Net, against four highly competitive approaches: CPS, CLD, GA-loss, and DMD. Figure 3 presents the quantitative results in terms of mean Dice score.

As illustrated, DuoMod-Net consistently and significantly outperforms the four competitive approaches across the evaluated tail classes. The performance gap is particularly pronounced in the more difficult categories. For instance, on the challenging duodenum class, our model achieves a mean Dice of 44.07%, representing an absolute margin of +7.95 percentage points over the leading competitor, GA-loss (36.12%). A similar trend is observed for the LAG, where our method (41.93%) surpasses the nearest competitor (GA-loss at 33.80%) by +8.13 percentage points. Even in more competitive classes such as the esophagus, our method (64.14%) maintains a notable lead over the second-highest method (GA-loss at 63.33%).

These improvements are statistically significant. As indicated by the annotations in Figure 3, our method’s performance advantage is demonstrated with ∗∗∗*p* < 0.001 for the vast majority of comparisons, ∗∗*p* < 0.01 for the comparison against DMD on the RAG class, and ∗*p* < 0.05 against GA-loss on the esophagus class. These results demonstrate the model’s effectiveness in addressing the inherent challenges of segmenting small, low-contrast, and anatomically variable structures, even under a limited 5% labeling regime.

### Ablation study of framework components

**Figure 4:**
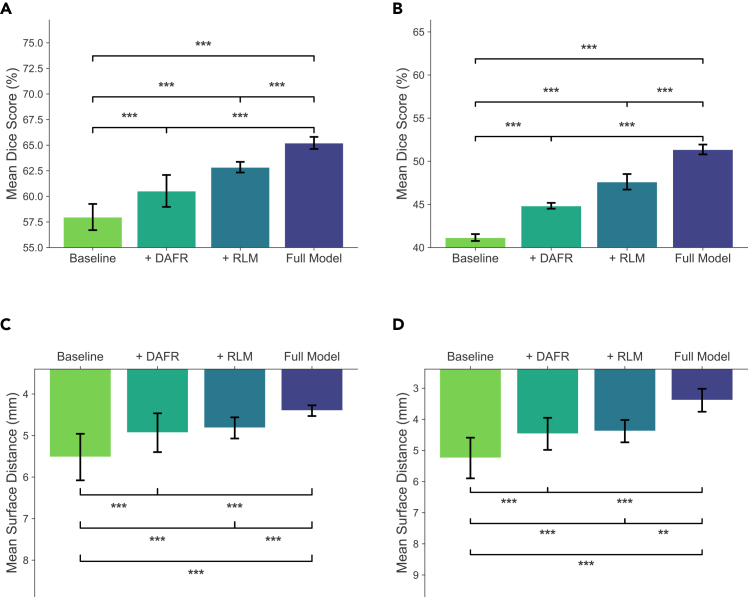
Ablation study demonstrating the effectiveness and synergy of the DAFR and RLM modules The study was performed on the AMOS dataset (5% labeled data setting) and evaluated four configurations: the baseline (CPS), the baseline with only DAFR (+DAFR), the baseline with only RLM (+RLM), and the full model combining both. Performance is shown for (A) overall foreground Dice, (B) tail-class Dice, (C) overall foreground average surface distance (ASD), and (D) tail-class ASD. For Dice scores (A and B), higher is better. For ASD (C and D), lower is better, and the *y* axis is inverted. Bar heights represent mean performance, and error bars indicate the standard deviation across three independent runs. Asterisks denote statistical significance from a paired Wilcoxon signed-rank test against the full model (∗∗p < 0.01 and ∗∗∗p < 0.001) after Benjamini-Hochberg correction.

To validate the individual contributions of our proposed DAFR and RLM modules, as well as their synergistic effect, we conducted an ablation study on the AMOS 5% labeled data setting. The results, summarized in Figure 4, demonstrate that both components are essential and complementary.

As shown in Figure 4, adding either the DAFR or the RLM module alone to the CPS baseline yields statistically significant improvements across the metrics (*p* < 0.001 vs. baseline). Focusing on the most challenging tail classes (Figures 4B and 4D), the RLM module provides the largest single-component improvement, increasing the average Dice_tail_ from 41.16% (baseline) to 47.62% and, correspondingly, reducing the average tail-class ASD from 5.24 to 4.38 mm.

Notably, the full model (DuoMod-Net), integrating both components, significantly outperforms both the baseline and each single-module configuration. It further elevates the average Dice_tail_ to 51.37%. This represents a statistically significant improvement even from the +RLM configuration (*p* < 0.01), indicating a distinct synergistic effect. This trend is mirrored in the surface distance, where the full model achieves the lowest tail-class ASD of 3.39 mm, a level not attained by either single-module configuration on its own.

Collectively, these results demonstrate that DAFR and RLM address different facets of the complex class-imbalance problem and are integral, complementary components of our proposed framework. The full set of detailed results for the metrics is available in Table S11.

### Mechanism analysis: Feature space dynamics

To validate the design rationale of the DAFR module—specifically the hypothesis that it expands the feature space during training and compresses it during inference—we performed principal-component analysis (PCA) and *L*_2_ norm analysis on feature embeddings. To capture the most informative dynamics, we specifically extracted the top-*k* hardest pixels (i.e., those exhibiting the highest modulation weights γ in Equation 1) from three representative tail classes (LAG, RAG, and gallbladder) and one head class (spleen) for comparison.

**Figure 5:**
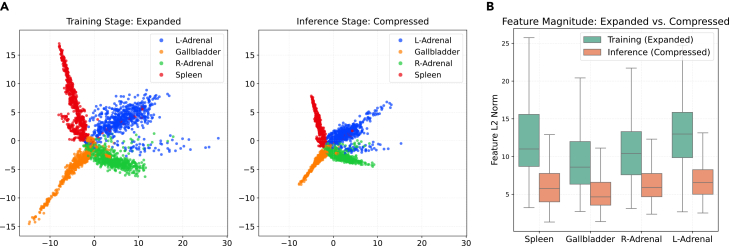
Visualization of feature space dynamics on the AMOS dataset under the 5% labeled setting (A) PCA projection of feature embeddings for the top-*k* hardest pixels (highest γ values). The left image shows the distribution during the training stage (with DAFR modulation), while the right image shows the same samples during the inference stage (without modulation). (B) Statistical analysis of feature *L*_2_ norms for the evaluated classes across the training and inference stages. Box bounds indicate the 25th and 75th percentiles, with the central line representing the median. Whiskers extend to the furthest data points within 1.5 times the interquartile range (IQR); outliers are omitted.

As illustrated in the PCA visualizations (Figure 5A), these hard samples exhibit a significantly dispersed and high-variance distribution in the feature space during the training stage. This dispersion is driven by the adaptive scaling factor γ, which intentionally pushes ambiguous features away from the decision boundary. Notably, when this modulation is removed at inference, the feature representations effectively collapse toward their respective class manifolds. This effect is visually most pronounced for the LAG (blue points), which transitions from a highly scattered cloud in the training phase to a tight, dense cluster during inference. This geometric convergence aligns with our quantitative findings, in which the LAG class demonstrated a notable performance lead (+8.13% Dice) over the closest competitor.

This observation is further quantified by the *L*_2_ norm distribution in Figure 5B, which shows a consistent reduction in feature magnitude from training to inference across the evaluated classes. This systematic compression suggests that the decision boundaries learned in the expanded training space establish a geometric safety margin for the unperturbed inference data. By effectively pulling the features away from the learned decision boundary during inference, the model naturally increases its classification margin and confidence. This geometric behavior distinguishes DAFR from simple random feature perturbations, demonstrating its role as a structured, uncertainty-driven geometric regularizer that enhances morphological recovery for difficult tail classes.

### Mechanism verification and sensitivity analysis

To validate the design rationale of our proposed modules and assess their robustness to hyperparameter variations, we conducted a comprehensive series of verification experiments. Detailed numerical results are provided in Tables S12–S15.

#### Mechanism verification: Directed refinement and logarithmic stability

We first investigated the source of DAFR’s efficacy to distinguish its contribution from unstructured feature perturbation. As shown in Table S12, while injecting random Gaussian noise (*σ* = 0.1,0.5) yields marginal gains, DAFR achieves a statistically significant improvement over the highest noise baseline (*p* < 0.001): it not only increases the overall Dice to 65.22% (vs. 62.26% with *σ* = 0.5) but, more importantly, raises the Dice_tail_ to 51.37% (vs. 46.75% with *σ* = 0.1). This indicates that the benefit is attributable to the directed nature of our disagreement-guided refinement—consistent with the geometric expansion dynamics analyzed in the mechanism analysis: feature space dynamics section—rather than stochastic feature perturbation. Similarly, for the RLM module, comparisons with linear weighting (62.74%) and CReST-style weighting^14^ (61.54%) demonstrate the effectiveness of our approach. Unlike linear schemes that often become aggressive or numerically unstable when facing the extreme imbalance typical in medical imaging, our logarithmic strategy effectively compresses the dynamic range. This allows for stable boosting of tail classes—improving the Dice_tail_ to 51.37% compared to 46.77% for linear weighting and 45.44% for CReST—while avoiding the optimization instability often associated with excessive linear penalties, resulting in improved overall performance (*p* < 0.001).

#### Hyperparameter sensitivity across datasets

We further analyzed the sensitivity of the framework to key hyperparameters.

• DAFR strength (τ): we evaluated the disagreement control parameter τ across both AMOS and WORD datasets (Table S13). On the AMOS dataset, the framework exhibits high stability: increasing τ from 1.0 to 2.0 yields negligible fluctuation in Dice (0.10% change), maintaining a high Dice_tail_ of 50.41%. This suggests that under strong anatomical constraints (as in AMOS), the feature space is robust enough to accommodate aggressive expansion. Conversely, on the WORD dataset, we observe a distinct performance ceiling: increasing τ to 2.0 leads to a statistically significant decrease in Dice (from 72.66% to 71.95%, *p* < 0.001). This disparity indicates that while geometric expansion is beneficial, excessive perturbation beyond an intrinsic semantic limit begins to disrupt class separability, causing feature manifolds to collide. At the lower end (*τ* = 0.1), performance consistently degrades across both datasets (e.g., dropping to 61.66% Dice on AMOS), suggesting that a sufficient perturbation amplitude is crucial for facilitating the escape from local minima. Collectively, these results delineate an effective operating window (*τ* ≈ 1.0) that is strong enough to trigger effective margin expansion yet constrained enough to respect the global semantic boundaries.
• RLM weight floor (*ω*_*floor*_): this parameter regulates the effective dynamic range of the weighting scheme. As analyzed in Table S14 under the 5% labeled AMOS setting, *ω*_*floor*_ = 0.05 is identified as the best-performing configuration, yielding the highest Dice of 65.22% and Dice_tail_ of 51.37%. Reducing the floor to 0.01 causes a notable performance drop (Dice decreases to 62.41%), suggesting that excessive suppression of easy classes can result in gradient starvation, where the model fails to maintain discriminative features for head classes. Conversely, raising the floor to 0.10 yields stable but slightly suboptimal results (Dice falls to 65.06% but Dice_tail_ to 49.93%), as it dilutes the corrective signal for tail classes.
• RLM percentile anchors: finally, Table S15 validates the rationale for our [5th,95th] percentile choice under the 5% labeled AMOS setting. Using the full range [0,100] exposes the normalization to transient mini-batch outliers, destabilizing the weighting stability and dropping Dice to 64.61% and Dice_tail_ to 49.71% (*p* < 0.001). Conversely, a narrower range [10,90] leads to excessive clipping, which collapses the valid inter-class variance and significantly reduces the discriminative signal (61.54% Dice; 48.47% Dice_tail_; *p* < 0.001). The [5,95] setting effectively filters statistical noise while preserving the essential frequency hierarchy, supporting its role as a robust and favorable operating point.

#### Computational efficiency

Regarding overhead, the calculation of Jensen-Shannon (JS) divergence and dynamic ranking increases per-iteration training time by approximately 19.0% (0.04 s increment from 0.21 to 0.25 s on an RTX 4090). This is a practical trade-off for the performance gains, and notably, inference speed remains unaffected since the modulation modules are deactivated during testing.

### Qualitative visualization

**Figure 6:**
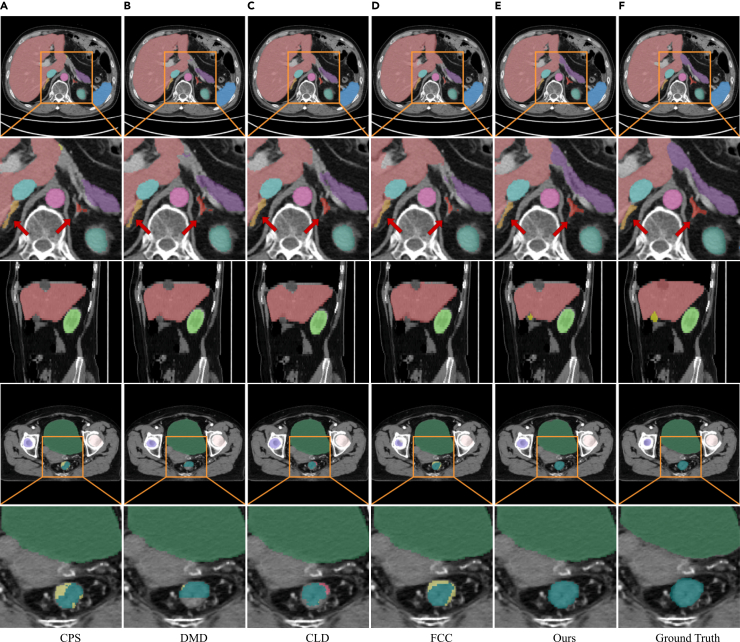
Qualitative comparison of segmentation results Rows 1–3 show examples from the AMOS dataset (5% labeled setting): adrenal glands (row 1, with magnified views in row 2) and gallbladder (row 3). Rows 4 and 5 show an example from the WORD dataset (5% labeled setting): rectum (row 4, with magnified views in row 5). Each column displays the predictions from a different evaluated method. Red arrows (row 2) explicitly indicate the locations of the adrenal glands.

To provide intuitive insights into the segmentation capabilities of our framework, DuoMod-Net, Figure 6 presents qualitative segmentation results for representative challenging cases from both the AMOS and WORD datasets. The first two rows focus on the adrenal glands (RAG: orange; LAG: red), small-volume structures in which competing methods (columns A–D) often exhibit fragmented predictions, significant inaccuracies, or complete omissions. DuoMod-Net (column E) not only accurately detects both glands but also demonstrates improved morphological fidelity, notably recovering the characteristic inverted Y-shape of the LAG (red), whereas other methods show discontinuous predictions or false positives lacking clear structure. Furthermore, these initial rows highlight the model’s ability to produce consistent and continuous segmentation of the elongated pancreas (purple), suggesting enhanced semantic continuity compared with fragmented results from competitors. Moving to the third row, DuoMod-Net accurately identifies the gallbladder (yellow), another challenging tail class due to its proximity to the liver, which is notably omitted by the other methods shown in this slice. Finally, the fourth and fifth rows, drawing examples from the WORD dataset, further illustrate the model’s consistent performance on challenging tail classes. DuoMod-Net correctly identifies the rectum (dark teal)—a highly challenging class due to its anatomical variability—and, as clearly shown in the magnified views in row 5, accurately captures its cross-sectional shape without the discontinuities or false positives (misclassifications as small intestine [pink] or colon [light yellow]) observed in competing results.

Collectively, these visual examples substantiate our quantitative findings, illustrating DuoMod-Net’s enhanced sensitivity to small-scale structures, improved capability in recovering morphology, and ability to maintain semantic continuity.

## Discussion

In this work, we introduced DuoMod-Net, a synergistic framework that integrates DAFR and RLM to address the persistent challenge of class-imbalanced semi-supervised medical image segmentation. Our extensive evaluation, detailed in the results section and the supplemental information, demonstrates the framework’s effectiveness and robustness through five principal findings. First, on in-domain benchmarks (AMOS and WORD datasets), our framework consistently achieves statistically significant improvements in the core volumetric metrics (Dice and Dice_tail_) compared to the competing methods (Table 1). Second, this performance advantage remains consistent across varying supervision levels; as demonstrated in our label-efficiency analysis, DuoMod-Net not only maintains its lead at 10% and 20% regimes but also significantly narrows the supervision gap, recovering over 90% of the fully supervised tail-class performance. Third, this advantage extends to the zero-shot, cross-dataset generalization test on FLARE22, particularly in tail-class performance (e.g., a 3.62% absolute gain in Dice_tail_). Fourth, our qualitative analysis (Figure 6) and reliability assessment (ASD^pen^) substantiate these gains, demonstrating our method’s ability to detect and recover the complex morphology of small-volume structures, such as the adrenal glands (RAG/LAG), and to maintain semantic continuity. Finally, our mechanistic verification and ablation studies (Figures 4 and 5) statistically and geometrically support our framework’s design, demonstrating that DAFR induces the proposed feature space expansion-compression dynamics (establishing a geometric safety margin) and that both modules yield a distinct synergistic effect (*p* < 0.01) with high stability against hyperparameter variations.

Together, these quantitative and qualitative findings establish our proposed framework as an effective and robust solution. To build upon these findings, we will first delve into the nuanced interpretations of our quantitative results. Following this, we will position our work within the broader context of foundation models and, finally, conclude with a discussion of the method’s limitations and avenues for future research.

### Geometric analysis and boundary behavior

A granular analysis of our evaluation metrics reveals important insights into the geometric behavior of our expansion-based strategy. While DuoMod-Net achieves leading performance in volumetric overlap (Dice), its boundary behavior and response to hyperparameter scaling present a nuanced picture. In this section, we untangle these geometric dynamics from three perspectives: first, we examine the interaction between our proactive expansion mechanism and the available anatomical label context (driving specific outliers on the WORD dataset); second, we address the inherent survivorship bias in tail-class surface distances; and finally, we abstract these observations to establish the theoretical limits of geometric perturbation, highlighting the synergistic stability between RLM and DAFR.

#### Impact of anatomical context on expansion

On the WORD dataset, our ASD (3.76 mm) was numerically higher than the compression-based method, FCC (3.55 mm). However, the per-class breakdown (Table S5) reveals that this is not a systemic degradation but is predominantly driven by outliers in the left kidney class (ASD: 12.1 ± 11.7 mm). This phenomenon stems from the interaction between our proactive expansion strategy and the ambiguity of lateral localization. Specifically, accurate segmentation of paired organs (e.g., kidneys) relies on distinguishing their laterality. In AMOS, major medial vessels (aorta and IVC) are labeled, serving as a distinct central reference frame that separates the left and right retroperitoneal spaces. In WORD, these landmarks are unlabeled background. Without these explicit cues to anchor spatial orientation, local patches of the left and right kidneys appear visually similar. Under this ambiguity, DAFR’s expansion mechanism—which aggressively seeks to recover potential false negatives—lacks the semantic constraints to suppress visually similar features on the opposite side. This can lead to contralateral confusion (e.g., predicting regions of the right kidney as left), resulting in significant distance penalties for these specific outliers. Notably, this hypothesis is supported by the AMOS results: when vascular landmarks are supervised, the spatial ambiguity regarding laterality is substantially mitigated. This effectively suppresses the severe outliers in the left kidney, as evidenced by our LK ASD stabilizing from 12.1 ± 11.7 mm in the WORD dataset to 5.0 ± 0.8 mm in AMOS. Consequently, without the burden of these substantial positional errors, DAFR achieves a lower overall ASD (4.40 vs. 4.70 mm for FCC). This suggests that adequate anatomical context effectively guides the expansion, significantly reducing the risk of severe boundary overshoot while maintaining high overall precision.

#### The survivorship bias in ASD

Furthermore, we consistently observed the counterintuitive phenomenon where tail-class surface distance metrics (ASD_tail_) appeared, on average, lower (i.e., better) than the overall foreground metrics (ASD). This observation stems from two concurrent factors. First, it reflects the inherent geometric constraint of small anatomical structures: tail classes (e.g., adrenal glands) have limited spatial extent. Consequently, even suboptimal segmentations—provided they are correctly localized—tend to produce bounded surface errors in absolute millimeters. Second, and critically, the standard ASD metric exhibits survivorship bias by excluding invalid values arising from complete detection failures. Since miss rates are significantly higher for tail classes, the reported ASD_tail_ effectively becomes a conditional metric, representing the accuracy of only the easiest instances that were successfully detected. This limitation motivates the ASD^pen^ analysis presented in the analysis of failure modes and boundary reliability section, which demonstrates that while baselines may achieve low ASD on detected tail organs, they exhibit high failure rates. In contrast, DuoMod-Net achieves an effective balance: it effectively minimizes catastrophic misses (lowest ASD^pen^) while maintaining high geometric precision on detected structures.

#### Geometric stability and theoretical limits

Finally, we address the theoretical relationship between our modules to explain the stability boundaries observed in our sensitivity analysis. While DAFR functions effectively as an independent geometric regularizer, our ablation results suggest that RLM serves as a synergistic gradient stabilizer. In a highly imbalanced regime, the feature space is often skewed by background dominance. By structurally mitigating this gradient bias, RLM provides a more balanced optimization landscape. As reflected in our stepwise ablation, this foundational stability enables DAFR to impose directed perturbations with greater overall efficacy. However, this capacity for geometric expansion is subject to an intrinsic theoretical limit. As evidenced by the performance drop at *τ* = 2.0 on the WORD dataset, pushing the perturbation strength beyond the effective window leads to degradation. This implies that if the expansion magnitude exceeds the intrinsic spatial margin between adjacent classes, their feature manifolds risk colliding and overlapping, which compromises semantic separability. Ultimately, these findings underscore the rationale for our default setting (*τ* ≈ 1.0), which effectively balances the need for proactive margin expansion against the preservation of intrinsic semantic boundaries.

### Positioning in the context of foundation models

It is essential to position our work within the current landscape of large-scale, generalist foundation models, such as TotalSegmentator,^27^ the Segment Anything Model (SAM),^28^ and SAM2,^29^ and their subsequent medical-specific adaptations.^30^^,^^31^^,^^32^^,^^33^ While these models represent a significant paradigm shift, our framework addresses a distinct, persistent, and practical challenge in clinical data analysis.

First, models such as TotalSegmentator are established, fully supervised frameworks trained on large-scale, diverse, and curated labeled datasets. Our work, in contrast, tackles the critical real-world problem of data scarcity and imbalance. We provide a systematic methodology for the common clinical scenario where acquiring thousands of expert-level, voxel-wise annotations is prohibitively expensive but large cohorts of unlabeled data are available. Furthermore, off-the-shelf tools such as TotalSegmentator are inherently fixed in their scope. A notable example is the rectum, which is defined as a distinct, under-represented tail class in the WORD dataset. In the TotalSegmentator model, the rectum is not available as a separate class but is merged into the general colon label. This presents a significant limitation for any clinical or research application requiring rectum-specific analysis (e.g., for rectal cancer staging). If a new, rare organ of interest is not part of its predefined 104-class set, TotalSegmentator cannot be easily adapted or retrained by the user. Our framework, conversely, is a methodology designed for such extensibility. It can be trained on any custom annotation set—including new, rare, or imbalanced structures such as the rectum—and our quantitative results (Table 1) and qualitative analysis (Figure 6) demonstrate its ability to effectively learn these specific tail classes.

Second, models like SAM, SAM2, and their medical variants remain fundamentally prompt-based, interactive tools. While powerful, their reliance on human-in-the-loop interaction (e.g., points and boxes) makes them unsuited for the high-throughput, fully automatic workflows required for clinical screening or large-scale data analysis. Our method, conversely, is designed to train a specialist model that operates automatically and reliably on a specific, predefined task (e.g., multi-organ segmentation), which is a key requirement for deployment in many clinical pipelines.

Finally, large-scale self-supervised pre-training (e.g., general-purpose methods such as MAE^34^ or DINO,^35^ as well as medical-specific approaches^36^^,^^37^^,^^38^^,^^39^^,^^40^) should be viewed as a synergistic ally rather than a direct competitor. Self-supervision provides an excellent mechanism for learning rich, general-purpose features from unlabeled data. Our framework, particularly the DAFR and RLM modules, can then serve as an effective and specialized fine-tuning strategy. One can envision a comprehensive pipeline in which a model is first pre-trained on a large unlabeled medical archive and then efficiently adapted to a specific, imbalanced, semi-supervised task using our proposed framework. Thus, our work provides a valuable component for translating the power of pre-training into robust, specialized clinical applications.

### Limitations and future directions

Despite the demonstrated performance and generalization capability of our framework, several limitations warrant discussion, which in turn motivate clear directions for future research.

First, regarding morphological complexity, our framework effectively recovers compact, under-represented objects by proactively expanding their decision regions. However, for highly variable and tortuous structures (e.g., the intestines), our uniform geometric perturbations do not inherently capture complex spatial topologies. As observed in our quantitative results on the WORD dataset, while our method maintains precise boundaries (low ASD) for these organs, it does not achieve the highest volumetric overlap (Dice). This indicates that our framework primarily addresses frequency-based imbalance rather than explicitly modeling complex anatomical shapes. Future work could integrate explicit topological priors—such as centerline constraints or persistent homology—directly into the semi-supervised optimization process to refine structural fidelity.

Second, regarding spatial context and laterality, as analyzed in the geometric analysis and boundary behavior section, our geometric expansion can be susceptible to symmetric ambiguity in landmark-sparse environments. When critical central reference frames (e.g., major retroperitoneal vessels) are unannotated, the model occasionally struggles to halt expansion, leading to contralateral confusion (as observed with the left kidney outliers). To resolve this lateral ambiguity in label-sparse datasets, future research should explore semi-supervised mechanisms for global relational reasoning. Incorporating explicit anatomical coordinate encodings or modeling inter-organ spatial relationships as a structural graph could provide the necessary macroscopic context without requiring fully annotated landmarks.

These limitations highlight a promising avenue for future work: moving from implicit geometric expansion to explicit structural and relational modeling. This will extend the effectiveness of our imbalance-correcting framework from compact objects to a broader spectrum of complex anatomical scenarios.

## Methods

### Problem formulation

We formally define the problem of CISSL in the context of medical image segmentation. Let the available data consist of a small labeled set $D^{l}={\left\{\left(x_{i},y_{i}\right)\right\}}_{i=1}^{n_{l}}$ and a large unlabeled set $D^{u}={\left\{\left(x_{j}\right)\right\}}_{j=1}^{n_{u}}$, where *n*_*l*_ ≪ *n*_*u*_. Here, $x\in R^{H\times W\times D}$ represents a 3D medical image, and *y* ∈ {0,1, …,*C*}^*H*×*W*×*D*^ is its corresponding pixel-wise annotation map with *C* foreground classes and one background class (indexed by 0). The class imbalance, characterized by a long-tailed distribution of voxel counts in $D^{l}$, is quantified by the imbalance ratio *ρ* = *n*_1_/*n*_*C*_, where *n*_*c*_ is the total voxel count for a foreground class *c*, and the classes are sorted by frequency such that *n*_1_ ≥ *n*_2_ ≥ … ≥ *n*_*C*_. While *ρ* is close to 1 in conventional SSL settings, in medical imaging it can be highly skewed (e.g., >400), indicating substantial class imbalance. Our objective is to train a segmentation network *f*_*θ*_, parameterized by θ, using both $D^{l}$ and $D^{u}$ to achieve consistent and balanced segmentation performance on unseen test sets with a similar long-tailed distribution, particularly for tail classes.

### Experimental protocol

Within the literature on class-imbalanced semi-supervised medical image segmentation, a common evaluation paradigm involves resampling the 3D volumes to a fixed, low-resolution grid (e.g., 80 × 160 × 160). While this approach simplifies inter-model comparison, it introduces a notable limitation: it distorts the true anatomical scale and discards high-frequency details essential for segmenting small, tail-class organs. More importantly, this fixed-grid paradigm deviates from clinical reality, where models must operate in a physically consistent space. To address these shortcomings and elevate the methodological rigor of the field, we introduce a standardized pipeline inspired by the established practices of nnU-Net.^41^ Specifically, the 3D volumes are resampled to a uniform physical spacing of 1.5 × 1.5 × 2.0 mm. This value is strategically chosen to reduce the physical anisotropy inherent in the raw median resolution (approximately 0.75 × 0.75 × 2.0 mm), thereby better aligning the anatomical structures with the isotropic 3 × 3 × 3 convolutional kernels of the V-Net backbone. Furthermore, this unified spacing is applied consistently across the datasets (AMOS, WORD, and FLARE22) to facilitate a standardized comparison. Inference is performed on these normalized volumes, and the resulting segmentations are resampled back to their original spacing for evaluation. Notably, to enable a consistent and rigorous comparison, we systematically re-implemented and benchmarked the competing methods within this unified pipeline. This rigorous approach ensures that any observed performance differences stem from intrinsic algorithmic differences rather than confounding variables such as data preprocessing or resolution sensitivity, a common issue in prior works.

The experiments were conducted on three public datasets. The AMOS dataset^9^ provides 360 scans, which we split into 216 for training, 24 for validation, and 120 for testing. The WORD dataset^10^ provides 150 scans, which we used according to its official split of 100 for training, 20 for validation, and 30 for testing. For zero-shot generalization testing, we used the 50 validation scans from the FLARE 2022 dataset.^11^ The experiments were conducted within the PyTorch framework (v.2.8.0) on NVIDIA RTX 3090 and 4090 GPUs. To facilitate a consistent comparison, the evaluated methods utilized a 3D V-Net as the segmentation backbone.^42^ We trained the networks for up to 500 epochs using an SGD optimizer with a momentum of 0.9 and a weight decay of 3 × 10^−5^. The initial learning rate was 0.03 and followed a poly decay schedule (power = 0.9). An early stopping mechanism with a patience of 50 epochs was employed, monitoring the mean foreground Dice score on the validation set. For data handling, we extracted random patches of size 64 × 128 × 128 during training and augmented them with random flipping. A specific preprocessing step was applied to the WORD dataset, in which the left (LHF) and right (RHF) heads of femoral classes were merged into a single class during training. These were subsequently separated back into LHF and RHF during inference via a centroid-based post-processing step. Each training batch was composed of half labeled and half unlabeled samples. The unsupervised loss weight, λ, was initialized at 0.1 and underwent a sigmoid ramp-up over the first 150 epochs. Regarding our proposed modules, the DAFR component was activated at epoch 100 with its inter-class strength (τ) set to 1.0. For the RLM module, *ω*_*floor*_ was adapted based on the imbalance severity of each dataset. Specifically, we set *ω*_*floor*_ = 0.05 for the highly imbalanced AMOS (*ρ* ≈ 407) to facilitate sufficient suppression of dominant classes while using a more conservative *ω*_*floor*_ = 0.20 for the less extreme WORD (*ρ* ≈ 125) to avoid over-suppression. In contrast, the weight ceiling (*ω*_ceiling_) was structurally fixed to 1.0 for all datasets. To ensure statistical reliability, the experiments were repeated three times using different random seeds (0, 1, or 2), and we report the mean and standard deviation of these runs. Finally, regarding the competing method DyCON,^24^ we encountered persistent numerical instability (e.g., loss divergence) during training across our datasets. For numerical stability and consistent comparisons, we integrated standard safeguards into its implementation, including gradient clipping (max norm 1.0), input clamping, and numerical stabilization for logarithmic operations. Despite these standard adaptations, the method remained unstable, leading to the observed variance in its reported performance.

### DAFR

**Figure 7:**
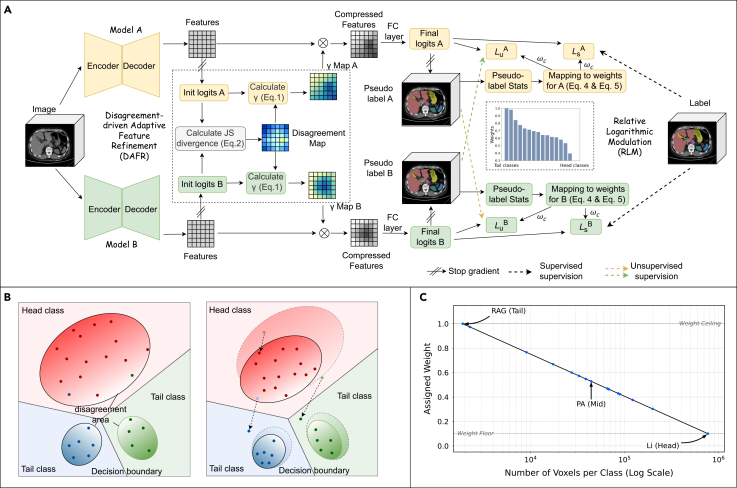
Overview of the proposed DuoMod-Net framework (A) The overall architecture builds upon the cross pseudo supervision (CPS)^6^ baseline by integrating the DAFR and RLM modules. (B) Conceptual illustration of the uncertainty-guided feature perturbation implemented via a train-test inversion protocol. During training (left diagram), features are amplified by γ to learn a robust boundary in an expanded space. At inference (right diagram), γ is removed, effectively compressing features relative to the boundary to enhance segmentation confidence. (C) Visualization of the relative logarithmic modulation (RLM) module, which dynamically computes class weights *ω*_*c*_ by anchoring to the foreground class distribution. This dual strategy tackles class imbalance from both feature and loss perspectives.

Our proposed framework, DuoMod-Net, builds upon the dual-model CPS^6^ baseline. We enhance this baseline by integrating two synergistic components: the DAFR module and the RLM module. The overall architecture is illustrated in Figure 7A.

To address the sparse features of tail classes, which are often encroached upon by the feature clusters of dominant classes, we introduce the DAFR module. Functionally, DAFR operates as a geometric regularization mechanism. By integrating theoretical principles from foundational adversarial training^43^^,^^44^ and recent feature perturbation strategies,^45^^,^^46^^,^^47^ it imposes structured regularization on the decision boundary. Unlike stochastic noise injection used in generic perturbation approaches, DAFR utilizes inter-model disagreement as a dynamic prior to selectively modulate feature magnitude.

Specifically, we generalize the geometric compression principle established in the FCC framework,^18^ transforming its static, class-wise formulation to an adaptive, voxel-level strategy. To realize this directed feature perturbation mechanism, we adopt a train-test inversion scheme, as illustrated conceptually in Figure 7B. During training, it acts as a specialist module that selectively amplifies feature magnitudes. Guided by Equation 1, this amplification primarily targets ambiguous samples from dominant (head) classes. This forces the classifier to learn a decision boundary that envelops an artificially expanded feature space. At inference, this amplification is removed. This retraction effectively compresses the feature representations relative to the learned boundary, establishing a geometric safety margin around the dominant classes. This reserved spatial latitude reduces encroachment on tail classes, conceptually paralleling contrastive refinement schemes in microscopy deblurring.^48^

This adaptive amplification is governed by a spatially adaptive scaling factor, *γ*^*j*^ ≥ 1. Its formulation synthesizes a class-level (inter-class) and a sample-level (intra-class) strategy:

(Equation 1) $$\gamma^{j}=1+\tau \cdot \underset{\text{inter}-\text{class}\;\text{term}}{\underset{︸}{\left(1-\frac{\text{rank}\left(p^{j}\right)}{C-1}\right)}}\cdot \underset{\text{intra}-\text{class}\;\text{term}}{\underset{︸}{\left({\hat{d}}^{j}\right)}}\text{,}$$

where τ is a hyperparameter controlling the overall refinement strength.

The inter-class term modulates the refinement intensity based on class dominance. Mathematically, this term implements a linear decay based on class rank, ensuring that classes with higher frequency (lower rank index, e.g., rank 0 for the most dominant class) receive the strongest feature amplification. This design aligns with our geometric regularization objective: since head classes possess the strongest gradients and densest clusters, they are most prone to over-occupying the feature space. By subjecting these dominant classes to higher γ values during training, we force the decision boundary to recede further from the class centers. Conversely, we deliberately attenuate this amplification for tail classes. Due to severe data scarcity, tail-class representations inherently exhibit high intra-class variance and spatial sparsity. Directly amplifying them (i.e., assigning high γ to high-rank classes) would primarily magnify intrinsic noise and outliers rather than legitimate semantic features, leading to decision boundary fragmentation rather than stable margin expansion. This proactive expansion penalizes tight boundaries, thereby reserving potential feature space that becomes accessible to under-represented tail classes during inference. The class rank, rank(*p*^*j*^), is dynamically determined using the global class distribution statistics.

The intra-class term, ${\hat{d}}^{j}$, quantifies sample-level uncertainty. It ensures that the expansion is not applied uniformly but focuses on samples lying near the decision boundaries. We leverage the disagreement between the two networks as a reliable proxy for this uncertainty. The raw disagreement, *d*^*j*^, is measured using the JS divergence between the predictive probability distributions, *P*_*A*_ and *P*_*B*_:

(Equation 2) $$d^{j}=\text{JS}\left(P_{A}\left(x^{j}\right)∥P_{B}\left(x^{j}\right)\right)\text{.}$$

To identify samples that are ambiguous relative to their peers, this raw disagreement is normalized using class-specific statistics tracked via an exponential moving average (EMA). The final intra-class term ${\hat{d}}^{j}$ is

(Equation 3) $${\hat{d}}^{j}=\frac{d^{j}-D_{\min,c}}{D_{\max,c}-D_{\min,c}+\epsilon },\text{where}\;c\;\text{is}\;\text{the}\;\text{pseudo}-\text{label}\;p^{j}$$

ϵ is a small constant (e.g., 1 × 10^−6^) added for numerical stability.

By synergizing these class-level and sample-level strategies, DAFR establishes a comprehensive regularization mechanism. The inter-class term acts as a global regulator that forces the decision boundary to leave space around dominant clusters, while the intra-class term functions as a local spotlight, selectively amplifying hard boundary samples to refine their structural delineations. This joint action effectively enlarges the classification margin for tail classes while enforcing a compact representation for high-confidence regions.

### RLM

To counteract biased gradients from class imbalance, we propose the RLM framework. While existing re-weighting schemes (e.g., class-balanced loss^49^) rely on absolute pixel frequencies, they suffer from an inherent limitation in medical segmentation: the background bias. The size of the background class is arbitrary, depending solely on the cropped field of view (FOV) rather than anatomical prevalence. Including this arbitrary background frequency in standard calculations collapses the relative weight differences among foreground organs. To address this, the first principle of RLM is foreground-background decoupling.

Second, even after decoupling the background, the foreground classes themselves exhibit a highly skewed long-tailed distribution, with frequencies spanning several orders of magnitude (e.g., from the liver to adrenal glands). This renders linear re-weighting schemes ineffective, as they often lead to gradient starvation for head classes or optimization instability for tail classes. The second principle of RLM is thus to operate in a relative, logarithmic domain. Unlike heuristic hard-mining approaches, RLM constructs a dynamic, frequency-adaptive normalization scale to normalize gradient contributions, as conceptually illustrated in Figure 7C.

RLM implements these principles via a systematic, multi-stage pipeline. First, a stable global class probability distribution, *π*, is maintained via an EMA. Notably, this calculation exclusively uses the foreground classes to determine the anchors for normalization.

#### Stable anchor selection (Winsorization)

Instead of using the absolute minimum or maximum frequencies—which are highly susceptible to statistical noise (e.g., artifacts) or initial training instability—we employ a percentile-based statistical approach. We explicitly clip the probability range to the 5th (*π*_5%_) and 95th (*π*_95%_) percentiles of the foreground class distribution. This strategy, akin to Winsorization statistics, effectively filters out transient outliers that would otherwise skew the gradient dynamic range. The probabilities are then clipped to this constrained range and transformed into a normalized value ${\hat{\pi }}_{c}\in \left[0,1\right]$ for each foreground class *c*:

(Equation 4) $${\hat{\pi }}_{c}=\frac{\log\left(\text{clip}\left(\pi_{c},\pi_{5\%},\pi_{95\%}\right)\right)-\log\left(\pi_{5\%}\right)}{\log\left(\pi_{95\%}\right)-\log\left(\pi_{5\%}\right)+\epsilon }\text{,}$$

where ϵ is a small constant (e.g., 1 × 10^−6^) added for numerical stability.

This equation normalizes the log probability of each foreground class relative to the log probabilities of the tail and head anchors (*π*_5%_ and *π*_95%_). A class with a probability near the tail anchor *π*_5%_ will have ${\hat{\pi }}_{c}$ close to 0, while a class near the head anchor *π*_95%_ will have ${\hat{\pi }}_{c}$ close to 1.

The final weight *ω*_*c*_ for each foreground class is then obtained by inverting and scaling this normalized value:

(Equation 5) $$\omega_{c}=\omega_{\text{floor}}+\left(1-{\hat{\pi }}_{c}\right)\cdot \left(\omega_{\text{ceiling}}-\omega_{\text{floor}}\right),\forall c\in \left\{1,\ldots ,C\right\}\text{,}$$

where [*ω*_floor_,*ω*_ceiling_] defines the target weight range.

#### Background pivot assignment

After the foreground weights are computed, the background class (*c* = 0) is assigned a stable weight, *ω*_0_, defined as the median of the foreground class weights. This design is critical: it establishes the background as a neutral pivot. By decoupling the background from the frequency-based ranking and assigning it the median foreground weight, we enable the model to maintain sufficient suppression of false positives while preventing the background area from dominating the gradient landscape. This effectively balances tail-class boosting with the maintenance of stable head-class features, preventing optimization collapse in highly imbalanced scenarios. Finally, the weights undergo mean normalization, and a separate, gentler set of weights for the Dice loss is generated by taking the square root of these primary weights.

## Resource availability

### Lead contact

Further information and requests for resources should be directed to and will be fulfilled by the lead contact, Prof. Shaohua Kevin Zhou (skevinzhou@ustc.edu.cn).

### Materials availability

This study did not generate new materials.

### Data and code availability

• All three benchmark datasets used in this study (AMOS,^9^ WORD,^10^ and FLARE 2022^11^) are publicly available online from their respective original sources.
• The original code and trained models generated in this study have been deposited at Zenodo: https://doi.org/10.5281/zenodo.19656480^50^ and are publicly available as of the date of publication. The source code is also maintained on GitHub at https://github.com/WayneBo98/DuoMod-Net.
• Any additional information required to reproduce or reanalyze the results reported in this paper is available from the lead contact upon request.

## Acknowledgments

This work is partially supported by the National 10.13039/501100001809Natural Science Foundation of China under grant 62271465, the 10.13039/501100012166National Key R&D Program of China under grant 2025YFC3408300, and the Suzhou Basic Research Program under grant SYG202338.

## Author contributions

W.B. contributed to the conceptualization, algorithm development, data processing, experiment conduction, data analysis, and manuscript writing. A.H. and Y.Z. contributed to manuscript review and editing. T.X. and Y.X. contributed to providing clinical insights and result validation. S.L. contributed to providing clinical insights, manuscript review, and supervision. S.K.Z. contributed to conceptualization, resources, manuscript review, and supervision. All authors have read and approved the manuscript.

## Declaration of interests

The authors declare no competing interests.

## Declaration of generative AI and AI-assisted technologies in the writing process

During the preparation of this work, the authors used Qwen from Alibaba in order to improve readability and language. After using this tool/service, the authors reviewed and edited the content as needed and take full responsibility for the content of the published article.

## Supplemental information

Document S1. Tables S1–S15 and supplemental methods

Document S2. Article plus supplemental information
