# Supplementary material for: DuoMod-Net: Logarithmic balancing and geometric refinement for imbalanced semi-supervised medical image segmentation
Source: Patterns (N Y). 2026 May 27;7(6):101570. doi: 10.1016/j.patter.2026.101570 (PMC13280726; doi:10.1016/j.patter.2026.101570)
Supplement: Document S1. Tables S1–S15 and supplemental methods [file mmc1.pdf]

Patterns, Volume *7*

Supplemental information

DuoMod-Net: Logarithmic balancing and geometric refinement for imbalanced semi-supervised medical image segmentation

Wang Bo, Along He, Ting Xue, Yue Zhang, Yi Xiao, Shiyuan Liu, and Shaohua Kevin Zhou

Supplemental Methods

Dataset class compositions

Below are the full lists of foreground classes for the three datasets used in our evaluation. The lists for AMOS and WORD are ordered by their approximate mean voxel counts (from largest to smallest) to illustrate the class imbalance.

- **WORD Dataset (16 Classes):** Liver (Li), Intestine (In), Colon (Co), Stomach (St), Bladder (Bl), Spleen (Sp), Left Kidney (LK), Right Kidney (RK), Right Head of Femur (RHF), Left Head of Femur (LHF), Pancreas (Pa), Duodenum (Du), Rectum (Re), Gallbladder (Ga), Esophagus (Es), Adrenal (Ad).
- **AMOS Dataset (15 Classes):** Liver (Li), Stomach (St), Spleen (Sp), Left Kidney (LK), Right Kidney (RK), Aorta (Ao), Bladder (Bl), Pancreas (Pa), Inferior Vena Cava (IVC), Duodenum (Du), Prostate/Uterus (P/U), Gallbladder (Ga), Esophagus (Es), Left Adrenal Gland (LAG), Right Adrenal Gland (RAG).
- **FLARE22 Dataset (13 Classes):** Contains 13 of the 15 AMOS labels, specifically excluding the Bladder (Bl) and Prostate/Uterus (P/U).

Definition of tail-class metrics

Throughout this paper, the tail-class metrics ($\text{Dice}_{\text{tail}}$ and ASD_{tail}) are calculated by averaging the respective metrics over a predefined set of challenging tail classes. This set must be common to both the training (AMOS) and generalization (FLARE22) datasets to ensure a fair evaluation of domain generalization.

For the AMOS dataset, the 6 smallest classes by voxel count are: RAG, LAG, Es, Ga, P/U, and Du. However, the FLARE22 dataset does not contain the Prostate/Uterus (P/U) label. Therefore, to construct a consistent evaluation set across domains, we exclude P/U and select the next smallest class present in both datasets (Duodenum). This results in the following 5-class set used for all `_tail` metrics on AMOS and FLARE22: Esophagus (Es), Gallbladder (Ga), Duodenum (Du), Right Adrenal Gland (RAG), and Left Adrenal Gland (LAG). For the WORD dataset, the tail-class definition is specific to its own label distribution (smallest 5 classes): Gallbladder (Ga), Esophagus (Es), Duodenum (Du), Adrenal (Ad), and Rectum (Re).

Table S1: Detailed per-class Mean Dice (%) on the AMOS dataset (5% labeled). Related to Table 1 and Figure 3.

Organs		Li	St	Sp	LK	RK	Ao	Bl	Pa	IVC	Du	P/U	Ga	Es	LAG	RAG
Imbalance ratio		407.2	105.0	64.4	47.2	45.1	36.0	34.6	23.3	20.4	17.3	14.4	9.2	4.8	1.2	1.0
V-Net (100%)		$95.6_{0.1}$	$88.0_{0.2}$	$93.4_{0.1}$	$93.2_{0.1}$	$92.8_{0.7}$	$91.0_{0.0}$	$88.0_{0.6}$	$81.2_{0.3}$	$86.7_{0.2}$	$73.8_{0.3}$	$81.2_{1.7}$	$75.6_{0.5}$	$77.7_{0.2}$	$66.2_{0.4}$	$66.2_{0.2}$
General	UA-MT	$85.5_{2.4}$	$51.3_{2.9}$	$75.9_{3.8}$	$70.0_{2.2}$	$71.3_{0.6}$	$78.5_{1.6}$	$47.6_{2.0}$	$46.2_{1.8}$	$63.7_{1.4}$	$26.4_{2.0}$	$30.9_{8.1}$	$47.2_{2.1}$	$52.1_{2.3}$	$30.5_{3.9}$	$39.7_{0.8}$
	CPS	$88.8_{0.2}$	$54.0_{3.5}$	$71.1_{6.3}$	$66.7_{4.8}$	$75.2_{2.2}$	$81.3_{2.0}$	$67.9_{3.1}$	$48.7_{1.9}$	$65.9_{0.9}$	$30.9_{2.3}$	$44.4_{4.0}$	$49.6_{0.4}$	$58.0_{2.3}$	$21.7_{3.2}$	$45.7_{1.3}$
	DST	$87.3_{1.0}$	$37.9_{1.5}$	$73.8_{2.1}$	$50.6_{5.0}$	$62.6_{3.0}$	$71.7_{3.9}$	$62.3_{3.0}$	$28.6_{3.3}$	$55.1_{3.8}$	$19.5_{4.4}$	$25.5_{8.6}$	$43.9_{2.3}$	$45.0_{6.4}$	$6.4_{9.0}$	$19.5_{14.2}$
	DMD	$86.9_{0.9}$	$52.4_{1.8}$	$79.0_{1.0}$	$75.8_{2.6}$	$75.8_{2.1}$	$82.5_{2.4}$	$70.7_{1.4}$	$45.9_{4.4}$	$65.7_{1.3}$	$33.6_{0.7}$	$49.4_{2.4}$	$51.7_{0.9}$	$60.5_{3.0}$	$33.2_{1.6}$	$47.4_{0.5}$
	SLCNet	$87.9_{1.0}$	$58.2_{1.3}$	$78.5_{1.1}$	$73.3_{6.5}$	$77.6_{1.6}$	$82.6_{1.4}$	$60.5_{8.1}$	$50.8_{1.6}$	$70.8_{1.4}$	$35.6_{5.5}$	$19.2_{0.0}$	$45.9_{1.4}$	$58.8_{1.2}$	$31.9_{2.0}$	$46.7_{2.8}$
	DyCON	$85.7_{1.4}$	$46.0_{3.0}$	$77.4_{1.8}$	$65.6_{3.6}$	$69.2_{1.7}$	$75.3_{1.6}$	$51.1_{4.2}$	$36.9_{7.7}$	$62.4_{3.1}$	$23.7_{4.0}$	$19.2_{0.0}$	$43.7_{2.3}$	$43.4_{2.1}$	$2.9_{4.1}$	$18.3_{6.8}$
Imbalanced	Adsh	$88.5_{0.4}$	$51.9_{2.9}$	$81.0_{2.2}$	$75.0_{2.2}$	$74.3_{1.6}$	$81.8_{1.1}$	$65.7_{1.6}$	$46.5_{2.9}$	$66.8_{4.1}$	$29.3_{5.2}$	$39.8_{4.2}$	$48.5_{1.1}$	$54.1_{2.4}$	$23.2_{2.5}$	$43.3_{3.8}$
	CLD	$90.2_{0.6}$	$62.2_{2.5}$	$79.2_{2.4}$	$68.8_{3.2}$	$77.5_{2.0}$	$82.6_{0.4}$	$65.4_{1.5}$	$55.5_{2.3}$	$69.5_{0.6}$	$35.4_{3.5}$	$48.7_{0.6}$	$50.5_{0.8}$	$57.1_{0.6}$	$30.2_{1.4}$	$47.3_{1.4}$
	DHC	$80.6_{1.3}$	$51.5_{3.8}$	$75.2_{2.1}$	$72.4_{1.2}$	$73.8_{1.9}$	$82.2_{1.6}$	$47.2_{6.2}$	$57.9_{0.4}$	$69.0_{0.7}$	$40.6_{1.1}$	$41.6_{6.4}$	$51.8_{0.9}$	$61.6_{1.8}$	$25.1_{2.6}$	$47.5_{1.8}$
	FCC	$88.5_{0.8}$	$57.8_{4.1}$	$76.2_{5.1}$	$74.7_{3.5}$	$78.1_{1.3}$	$75.8_{1.2}$	$71.9_{1.6}$	$54.2_{2.8}$	$61.7_{0.9}$	$26.7_{0.9}$	$49.8_{0.8}$	$46.5_{0.7}$	$58.1_{1.7}$	$27.0_{3.0}$	$46.2_{0.7}$
	GA-loss	$88.1_{0.9}$	$57.9_{4.4}$	$81.2_{1.1}$	$74.6_{3.6}$	$76.2_{1.0}$	$84.4_{0.6}$	$64.8_{7.1}$	$54.0_{4.9}$	$74.5_{2.2}$	$36.1_{5.8}$	$43.6_{2.7}$	$51.7_{0.3}$	$63.3_{2.9}$	$33.8_{5.0}$	$47.2_{3.1}$
	SKCDF	$88.5_{0.2}$	$54.3_{1.9}$	$75.1_{3.0}$	$62.5_{6.4}$	$72.4_{2.2}$	$79.5_{0.6}$	$59.5_{0.2}$	$48.2_{3.1}$	$64.6_{2.0}$	$31.3_{2.5}$	$46.0_{4.0}$	$46.9_{2.1}$	$53.9_{1.0}$	$30.3_{1.7}$	$44.5_{2.4}$
	Ours	$81.9_{0.9}$	$57.4_{1.8}$	$79.2_{2.3}$	$79.2_{3.7}$	$78.8_{0.5}$	$85.1_{0.7}$	$70.1_{2.1}$	$61.7_{1.6}$	$74.0_{1.3}$	$44.1_{1.3}$	$54.0_{0.1}$	$57.1_{1.4}$	$64.1_{0.8}$	$41.9_{2.3}$	$49.6_{0.8}$

Notes: Results are presented as mean_{std} over three independent runs. Full organ abbreviations are defined in the Supplemental Methods section. The imbalance ratio is relative to the voxel count of the smallest class (RAG).

Table S2: Detailed per-class Mean Dice (%) on the WORD dataset (5% labeled). Related to Table 1.

	Organs	Li	In	Co	St	Bl	Sp	LK	RK	RHF	LHF	Pa	Du	Re	Ga	Es	Ad
	Ratio	125.5	97.1	71.4	41.3	24.5	21.5	15.5	14.9	14.1	13.9	8.3	7.2	6.1	1.6	1.4	1.0
	V-Net	$95.5_{0.0}$	$85.9_{0.1}$	$83.5_{0.2}$	$90.0_{0.1}$	$90.4_{0.6}$	$93.3_{0.2}$	$92.7_{0.9}$	$93.4_{0.5}$	$91.4_{0.1}$	$90.5_{0.1}$	$81.6_{0.4}$	$64.9_{0.7}$	$79.3_{0.7}$	$77.4_{0.2}$	$72.5_{0.2}$	$64.7_{0.1}$
General	UA-MT	$88.1_{2.1}$	$27.7_{15.4}$	$14.1_{5.1}$	$48.6_{3.3}$	$39.2_{4.9}$	$68.3_{5.6}$	$65.1_{0.8}$	$65.6_{1.7}$	$85.7_{2.0}$	$83.3_{1.1}$	$52.4_{1.9}$	$26.1_{1.6}$	$4.7_{2.1}$	$31.1_{9.2}$	$26.4_{7.5}$	$27.8_{2.9}$
	CPS	$93.2_{0.1}$	$75.6_{1.0}$	$72.2_{0.2}$	$77.6_{1.0}$	$80.4_{0.8}$	$77.0_{1.4}$	$75.0_{1.5}$	$76.9_{0.5}$	$89.8_{0.3}$	$87.6_{0.3}$	$62.4_{1.0}$	$46.2_{1.7}$	$63.6_{1.4}$	$62.5_{1.8}$	$54.4_{1.0}$	$46.3_{3.0}$
	DST	$92.5_{0.2}$	$72.8_{1.4}$	$71.0_{0.3}$	$76.9_{0.9}$	$78.9_{1.1}$	$74.2_{1.1}$	$66.9_{1.7}$	$74.3_{0.2}$	$79.2_{3.4}$	$77.9_{4.6}$	$58.7_{1.4}$	$44.9_{1.2}$	$61.4_{1.2}$	$59.5_{6.1}$	$51.5_{3.2}$	$18.7_{16.9}$
	DMD	$93.0_{0.6}$	$73.4_{0.9}$	$72.0_{0.2}$	$77.9_{0.5}$	$82.9_{0.7}$	$76.3_{1.6}$	$74.7_{0.7}$	$76.5_{0.7}$	$89.8_{0.5}$	$87.8_{1.0}$	$63.6_{0.3}$	$46.9_{1.0}$	$62.5_{1.4}$	$61.7_{0.4}$	$55.0_{1.9}$	$45.5_{1.4}$
	SLCNet	$91.9_{0.8}$	$73.2_{1.3}$	$67.2_{1.1}$	$77.9_{0.9}$	$80.9_{2.5}$	$82.9_{1.2}$	$74.5_{0.2}$	$81.1_{4.1}$	$88.4_{1.5}$	$84.5_{2.2}$	$65.6_{1.6}$	$48.6_{1.7}$	$56.0_{1.5}$	$53.5_{4.8}$	$55.6_{1.7}$	$46.0_{2.5}$
	DyCON	$77.9_{6.1}$	$2.9_{1.8}$	$1.8_{1.4}$	$29.1_{6.3}$	$10.2_{12.9}$	$59.6_{8.5}$	$49.0_{11.3}$	$51.5_{12.2}$	$82.5_{3.4}$	$77.6_{4.9}$	$39.9_{12.4}$	$7.4_{7.9}$	$1.3_{0.8}$	$4.3_{3.2}$	$12.8_{12.3}$	$0.6_{0.9}$
Imbalanced	Adsh	$93.3_{0.1}$	$74.3_{1.2}$	$71.6_{1.0}$	$79.2_{0.6}$	$82.9_{1.0}$	$77.0_{0.8}$	$73.9_{1.5}$	$74.9_{1.0}$	$89.0_{1.1}$	$87.8_{1.2}$	$63.7_{2.0}$	$46.8_{1.2}$	$60.3_{2.0}$	$63.2_{0.7}$	$57.3_{0.4}$	$43.7_{0.8}$
	CLD	$93.0_{0.6}$	$75.9_{0.9}$	$72.3_{0.8}$	$79.1_{1.1}$	$80.4_{1.4}$	$76.3_{2.9}$	$77.2_{0.2}$	$78.0_{0.6}$	$90.0_{0.7}$	$88.2_{0.4}$	$64.5_{0.9}$	$47.7_{1.8}$	$61.9_{2.7}$	$63.0_{2.0}$	$56.9_{1.4}$	$45.1_{2.2}$
	DHC	$87.4_{2.5}$	$65.0_{5.6}$	$59.2_{2.9}$	$71.0_{2.7}$	$76.2_{1.3}$	$72.7_{4.3}$	$63.1_{11.6}$	$67.2_{8.5}$	$72.5_{17.0}$	$62.0_{23.1}$	$56.5_{4.4}$	$38.4_{2.7}$	$37.4_{26.5}$	$58.2_{1.8}$	$52.7_{2.2}$	$45.0_{3.7}$
	FCC	$92.4_{0.3}$	$76.1_{0.7}$	$72.8_{0.9}$	$78.7_{1.1}$	$81.0_{1.5}$	$76.5_{0.2}$	$75.6_{0.4}$	$78.7_{1.8}$	$88.5_{0.5}$	$86.9_{0.9}$	$62.4_{1.0}$	$47.7_{1.4}$	$60.9_{2.5}$	$63.9_{1.1}$	$55.9_{1.3}$	$47.4_{0.7}$
	GA-loss	$93.2_{0.1}$	$74.0_{0.2}$	$71.3_{0.3}$	$78.3_{0.9}$	$80.4_{0.9}$	$77.4_{1.2}$	$73.8_{0.7}$	$75.6_{0.4}$	$87.5_{0.7}$	$84.7_{0.6}$	$64.7_{0.3}$	$45.6_{1.3}$	$62.9_{0.9}$	$64.2_{0.7}$	$53.4_{1.5}$	$45.3_{0.5}$
	SKCDF	$93.2_{0.5}$	$74.4_{2.2}$	$71.9_{0.8}$	$76.8_{0.5}$	$80.4_{0.3}$	$78.8_{0.7}$	$74.8_{2.1}$	$76.5_{2.7}$	$88.1_{1.0}$	$85.9_{0.6}$	$62.6_{0.9}$	$43.9_{2.0}$	$61.3_{1.7}$	$63.1_{0.9}$	$55.5_{2.1}$	$47.5_{1.0}$
	Ours	$93.0_{0.2}$	$73.7_{1.1}$	$72.1_{0.5}$	$78.4_{1.1}$	$83.9_{0.1}$	$79.6_{1.8}$	$77.7_{0.6}$	$78.4_{0.0}$	$90.0_{0.2}$	$88.2_{0.5}$	$65.8_{1.0}$	$47.8_{0.9}$	$65.0_{1.5}$	$64.6_{0.6}$	$56.2_{0.9}$	$48.3_{1.8}$

Notes: Results are presented as mean_{std} over three independent runs. Full organ abbreviations are defined in the Supplemental Methods. The 'Ratio' row indicates the class imbalance ratio relative to the voxel count of the smallest class (Ad). V-Net serves as the fully supervised (100% labels) performance upper bound.

Table S3: Detailed per-class Mean Dice (%) on the FLARE22 dataset (zero-shot test). Related to Table 1.

Organs		Li	St	Sp	LK	RK	Ao	IVC	Pa	Du	Ga	Es	LAG	RAG
Imbalance ratio		389.5	76.1	59.6	58.7	54.1	21.3	20.3	20.1	17.3	5.2	3.6	1.8	1.0
V-Net (100%)		$94.8_{0.2}$	$85.3_{0.4}$	$88.9_{0.6}$	$85.0_{0.8}$	$83.8_{1.1}$	$89.0_{0.1}$	$83.5_{0.4}$	$78.6_{0.7}$	$65.0_{0.7}$	$69.0_{3.6}$	$75.0_{0.5}$	$63.8_{0.7}$	$66.1_{1.0}$
General	UA-MT	$86.9_{0.9}$	$46.9_{2.1}$	$66.6_{2.3}$	$64.6_{3.9}$	$63.1_{1.8}$	$81.7_{0.9}$	$64.6_{1.0}$	$35.9_{2.6}$	$17.5_{1.1}$	$44.2_{0.4}$	$51.5_{2.9}$	$23.7_{1.6}$	$41.3_{2.7}$
	CPS	$87.9_{1.0}$	$45.8_{5.5}$	$50.7_{9.5}$	$53.6_{3.9}$	$61.0_{1.0}$	$84.2_{1.9}$	$60.5_{1.5}$	$30.5_{5.4}$	$14.7_{2.2}$	$40.0_{3.3}$	$55.3_{4.0}$	$10.9_{1.4}$	$40.2_{5.9}$
	DST	$87.8_{0.5}$	$25.4_{3.0}$	$59.5_{4.2}$	$30.6_{7.5}$	$46.7_{6.7}$	$77.4_{3.3}$	$50.5_{7.9}$	$10.9_{3.3}$	$8.6_{4.1}$	$33.2_{7.6}$	$40.2_{6.7}$	$4.3_{3.6}$	$17.0_{11.4}$
	DMD	$85.2_{0.8}$	$45.4_{2.4}$	$62.7_{1.7}$	$56.8_{3.6}$	$56.5_{2.4}$	$83.9_{2.8}$	$59.6_{1.9}$	$20.4_{3.9}$	$17.0_{2.0}$	$37.8_{1.2}$	$55.3_{4.2}$	$24.9_{1.3}$	$42.6_{2.9}$
	SLCNet	$89.1_{1.3}$	$57.7_{0.6}$	$68.0_{2.1}$	$58.6_{6.4}$	$65.4_{0.8}$	$84.4_{1.3}$	$66.6_{3.3}$	$35.0_{4.0}$	$23.6_{8.4}$	$32.5_{2.0}$	$57.0_{1.0}$	$22.1_{3.4}$	$48.0_{2.5}$
	DyCON	$83.8_{1.7}$	$43.1_{2.9}$	$68.0_{1.5}$	$59.7_{6.3}$	$59.4_{2.5}$	$74.9_{1.9}$	$58.7_{2.7}$	$30.4_{9.9}$	$14.9_{3.1}$	$38.4_{3.8}$	$41.6_{8.5}$	$5.2_{1.7}$	$22.8_{7.6}$
Imbalanced	Adsh	$85.8_{2.0}$	$43.4_{1.9}$	$65.1_{1.4}$	$52.2_{3.0}$	$55.7_{4.4}$	$84.0_{1.0}$	$59.3_{4.3}$	$27.8_{2.1}$	$16.2_{4.8}$	$39.0_{9.5}$	$54.1_{1.9}$	$16.4_{1.2}$	$38.7_{7.2}$
	CLD	$88.6_{0.0}$	$53.3_{2.3}$	$57.4_{5.6}$	$44.8_{7.5}$	$64.0_{1.5}$	$84.0_{0.2}$	$66.1_{1.8}$	$36.5_{4.8}$	$19.6_{1.5}$	$38.8_{2.1}$	$56.1_{1.0}$	$20.5_{0.6}$	$45.8_{0.9}$
	DHC	$79.2_{1.1}$	$43.0_{7.4}$	$61.0_{3.4}$	$54.7_{4.2}$	$60.8_{0.8}$	$80.9_{2.3}$	$61.5_{3.1}$	$42.3_{2.1}$	$25.4_{3.9}$	$41.0_{1.3}$	$61.2_{3.1}$	$16.3_{1.9}$	$39.5_{4.3}$
	FCC	$86.2_{1.3}$	$47.1_{7.6}$	$55.2_{5.4}$	$57.0_{5.5}$	$63.2_{0.6}$	$78.3_{2.5}$	$52.4_{3.5}$	$34.6_{4.1}$	$12.9_{0.5}$	$38.7_{2.7}$	$55.0_{1.4}$	$15.8_{4.5}$	$40.3_{1.0}$
	GA-loss	$86.8_{0.5}$	$47.8_{6.5}$	$66.5_{1.0}$	$63.8_{5.2}$	$66.4_{2.2}$	$85.7_{0.5}$	$69.2_{2.1}$	$37.4_{8.5}$	$20.2_{4.3}$	$47.8_{2.1}$	$60.8_{3.5}$	$26.7_{3.4}$	$44.0_{4.5}$
	SKCDF	$88.2_{0.7}$	$47.6_{5.5}$	$57.8_{2.1}$	$48.2_{8.5}$	$57.6_{0.7}$	$81.2_{0.6}$	$63.1_{2.8}$	$30.0_{5.1}$	$17.1_{2.0}$	$31.8_{3.6}$	$53.5_{1.7}$	$20.2_{2.7}$	$42.3_{1.3}$
	Ours	$78.3_{1.3}$	$52.6_{1.6}$	$60.1_{4.9}$	$61.1_{6.7}$	$64.2_{2.0}$	$86.5_{0.5}$	$68.8_{2.6}$	$46.4_{2.7}$	$26.6_{1.0}$	$47.5_{2.6}$	$62.7_{1.0}$	$34.9_{2.2}$	$46.0_{0.4}$

Notes: Results are presented as mean_{std} over three independent runs. Full organ abbreviations are defined in the Supplemental Methods section. The imbalance ratio is relative to the voxel count of the smallest class (RAG).

Table S4: Detailed per-class Mean Average Surface Distance (ASD, mm) on the AMOS dataset (5% labeled). Related to Table 1.

Organs		Li	St	Sp	LK	RK	Ao	Bl	Pa	IVC	Du	P/U	Ga	Es	LAG	RAG
Imbalance ratio		407.2	105.0	64.4	47.2	45.1	36.0	34.6	23.3	20.4	17.3	14.4	9.2	4.8	1.2	1.0
V-Net (100%)		$1.0_{0.1}$	$1.7_{0.5}$	$0.9_{0.3}$	$2.2_{0.5}$	$1.8_{0.8}$	$1.9_{0.1}$	$1.2_{0.1}$	$1.2_{0.1}$	$0.9_{0.1}$	$3.1_{0.1}$	$1.2_{1.7}$	$1.1_{0.7}$	$1.9_{0.1}$	$1.4_{0.1}$	$0.9_{0.1}$
General	UA-MT	$4.4_{2.3}$	$6.9_{0.3}$	$9.6_{3.4}$	$12.2_{2.0}$	$6.9_{0.6}$	$3.2_{0.1}$	$22.8_{6.5}$	$7.2_{0.2}$	$6.4_{3.9}$	$11.4_{1.4}$	$9.7_{4.9}$	$14.2_{4.3}$	$4.0_{0.4}$	$9.2_{3.2}$	$4.1_{0.5}$
	CPS	$2.2_{0.1}$	$8.2_{1.3}$	$3.8_{0.5}$	$6.7_{0.9}$	$7.0_{1.9}$	$1.7_{0.5}$	$6.1_{2.5}$	$6.4_{1.1}$	$2.9_{0.4}$	$7.5_{0.5}$	$11.4_{1.6}$	$6.7_{2.8}$	$2.7_{0.4}$	$4.9_{0.2}$	$2.9_{0.2}$
	DST	$1.8_{0.4}$	$12.1_{0.5}$	$6.0_{1.6}$	$9.4_{2.6}$	$9.2_{7.0}$	$3.1_{0.7}$	$11.6_{2.0}$	$13.6_{3.1}$	$6.0_{3.2}$	$11.3_{1.9}$	$10.9_{12.9}$	$11.8_{2.5}$	$5.5_{1.5}$	$122.6_{104.3}$	$4.2_{0.8}$
	DMD	$1.8_{0.3}$	$7.5_{0.5}$	$3.4_{0.2}$	$4.5_{0.8}$	$4.9_{0.7}$	$1.6_{0.4}$	$8.8_{2.7}$	$6.3_{0.8}$	$2.7_{0.3}$	$6.6_{0.6}$	$10.6_{0.7}$	$5.6_{0.8}$	$2.4_{0.4}$	$3.5_{0.6}$	$2.4_{0.1}$
	SLCNet	$1.7_{0.1}$	$9.3_{3.3}$	$6.2_{0.4}$	$8.1_{2.6}$	$6.7_{1.0}$	$2.6_{0.8}$	$12.3_{6.4}$	$5.7_{0.7}$	$2.5_{1.0}$	$8.0_{0.9}$	-	$11.6_{3.8}$	$3.2_{0.6}$	$6.1_{1.5}$	$3.8_{1.2}$
	DyCON	$2.1_{0.4}$	$10.8_{0.6}$	$7.5_{2.1}$	$9.7_{1.8}$	$8.0_{2.2}$	$6.1_{2.5}$	$23.2_{7.3}$	$7.9_{1.3}$	$5.0_{1.7}$	$11.1_{2.4}$	-	$15.4_{4.3}$	$4.9_{0.2}$	$7.8_{0.0}$	$8.2_{3.8}$
Imbalanced	Adsh	$1.4_{0.2}$	$7.9_{0.5}$	$3.9_{0.6}$	$6.0_{0.8}$	$4.2_{1.2}$	$1.7_{0.2}$	$10.3_{1.7}$	$6.7_{0.8}$	$2.6_{0.6}$	$7.8_{1.3}$	$9.9_{0.5}$	$8.5_{0.3}$	$2.8_{0.3}$	$4.9_{0.2}$	$2.8_{0.4}$
	CLD	$1.6_{0.2}$	$5.3_{0.4}$	$2.4_{0.3}$	$4.8_{0.2}$	$7.2_{1.7}$	$1.5_{0.1}$	$8.5_{2.8}$	$4.7_{0.4}$	$1.8_{0.1}$	$6.8_{0.7}$	$10.8_{0.1}$	$6.8_{1.8}$	$2.7_{0.1}$	$3.4_{0.5}$	$2.6_{0.5}$
	DHC	$8.5_{0.7}$	$8.7_{0.5}$	$11.8_{3.5}$	$9.5_{1.9}$	$5.2_{0.9}$	$2.3_{0.9}$	$14.7_{5.3}$	$5.7_{0.9}$	$2.6_{0.5}$	$7.3_{0.4}$	$12.5_{1.4}$	$6.2_{1.4}$	$2.6_{0.7}$	$6.2_{1.2}$	$2.8_{0.6}$
	FCC	$2.5_{0.4}$	$6.4_{0.9}$	$3.0_{0.4}$	$4.4_{0.1}$	$5.2_{0.7}$	$2.6_{0.4}$	$4.8_{1.2}$	$4.8_{0.4}$	$2.9_{0.3}$	$8.3_{0.3}$	$11.5_{0.9}$	$4.4_{0.4}$	$2.6_{0.1}$	$3.8_{0.3}$	$2.4_{0.2}$
	GA-loss	$1.8_{0.2}$	$7.0_{1.3}$	$4.5_{0.1}$	$7.8_{2.0}$	$7.8_{0.8}$	$1.6_{0.3}$	$4.4_{0.7}$	$5.0_{1.1}$	$1.9_{0.8}$	$6.6_{0.9}$	$10.0_{1.0}$	$7.7_{1.2}$	$1.7_{0.5}$	$4.1_{0.6}$	$2.6_{0.3}$
	SKCDF	$2.0_{0.2}$	$9.3_{1.1}$	$5.5_{0.7}$	$10.7_{2.7}$	$8.2_{2.4}$	$3.0_{0.3}$	$15.3_{4.0}$	$8.3_{1.7}$	$3.7_{1.0}$	$9.5_{0.8}$	$12.7_{1.1}$	$13.8_{2.8}$	$3.7_{0.6}$	$7.5_{1.3}$	$4.0_{0.2}$
	Ours	$2.8_{0.4}$	$7.5_{0.4}$	$4.1_{0.5}$	$5.0_{0.8}$	$4.7_{1.1}$	$1.1_{0.1}$	$8.4_{1.0}$	$4.0_{0.3}$	$1.2_{0.2}$	$4.9_{0.5}$	$10.2_{0.4}$	$4.5_{1.3}$	$1.8_{0.2}$	$3.2_{0.7}$	$2.3_{0.0}$

Notes: Results are presented as mean_{std} over three independent runs. Full organ abbreviations are defined in the Supplemental Methods section. The imbalance ratio is relative to the voxel count of the smallest class (RAG). Entries marked with "-" indicate invalid surface distance calculations due to prediction collapse. Values such as 122.6 mm denote catastrophic detection failures with large false-positive outliers.

Table S5: Detailed per-class Mean Average Surface Distance (ASD, mm) on the WORD dataset (5% labeled). Related to Table 1.

	Organs	Li	In	Co	St	Bl	Sp	LK	RK	RHF	LHF	Pa	Du	Re	Ga	Es	Ad
	Ratio	125.5	97.1	71.4	41.3	24.5	21.5	15.5	14.9	14.1	13.9	8.3	7.2	6.1	1.6	1.4	1.0
	V-Net	$0.2_{0.0}$	$0.6_{0.0}$	$1.6_{0.0}$	$0.6_{0.1}$	$0.4_{0.1}$	$0.4_{0.1}$	$0.3_{0.1}$	$0.5_{0.4}$	$0.3_{0.0}$	$1.7_{0.8}$	$0.8_{0.1}$	$2.9_{0.1}$	$1.2_{0.5}$	$1.1_{0.2}$	$0.8_{0.0}$	$1.0_{0.0}$
General	UA-MT	$3.2_{2.0}$	$11.3_{5.2}$	$14.4_{2.4}$	$29.2_{6.7}$	$38.1_{3.5}$	$6.7_{1.8}$	$18.3_{2.6}$	$16.2_{3.3}$	$0.6_{0.3}$	$8.9_{3.0}$	$4.9_{1.1}$	$11.9_{0.8}$	$30.7_{11.6}$	$17.6_{11.5}$	$7.9_{1.8}$	$8.4_{1.8}$
General	CPS	$0.8_{0.1}$	$1.9_{0.1}$	$3.6_{0.0}$	$2.8_{0.1}$	$11.5_{3.8}$	$4.8_{2.5}$	$4.2_{0.4}$	$4.0_{1.1}$	$0.4_{0.0}$	$5.1_{0.7}$	$3.2_{1.0}$	$4.9_{0.3}$	$3.5_{1.0}$	$3.9_{0.3}$	$2.4_{0.1}$	$3.9_{0.7}$
General	DST	$1.7_{0.9}$	$2.1_{0.3}$	$3.7_{0.1}$	$3.2_{0.6}$	$6.5_{2.4}$	$12.7_{14.0}$	$6.7_{3.6}$	$11.8_{8.8}$	$0.8_{0.1}$	$6.1_{2.9}$	$3.7_{1.2}$	$5.4_{0.2}$	$5.9_{2.0}$	$3.4_{0.6}$	$3.1_{0.7}$	$58.7_{54.0}$
General	DMD	$0.7_{0.1}$	$1.9_{0.0}$	$3.6_{0.1}$	$2.7_{0.1}$	$9.0_{2.5}$	$6.0_{2.9}$	$6.9_{3.8}$	$2.7_{0.2}$	$0.4_{0.0}$	$5.4_{1.5}$	$3.0_{0.1}$	$4.6_{0.1}$	$4.4_{1.2}$	$3.8_{0.5}$	$2.6_{0.4}$	$3.6_{0.2}$
General	SLCNet	$1.0_{0.1}$	$2.3_{0.2}$	$4.3_{0.2}$	$3.0_{1.1}$	$10.2_{6.7}$	$4.1_{1.8}$	$16.4_{8.7}$	$2.9_{0.7}$	$0.4_{0.1}$	$8.4_{2.1}$	$3.0_{0.7}$	$5.2_{0.2}$	$5.0_{1.4}$	$3.9_{0.2}$	$3.1_{0.2}$	$3.1_{0.6}$
General	DyCON	$2.5_{0.3}$	$43.6_{11.8}$	$46.4_{6.0}$	$60.5_{31.1}$	$99.9_{16.0}$	$23.0_{13.3}$	$12.3_{6.1}$	$12.3_{4.0}$	$0.8_{0.3}$	$8.7_{3.0}$	$10.5_{2.2}$	$25.9_{7.5}$	$52.7_{29.0}$	$31.2_{3.6}$	$18.0_{9.0}$	$16.0_{0.0}$
Imbalanced	Adsh	$0.8_{0.1}$	$1.9_{0.1}$	$3.6_{0.1}$	$2.7_{0.1}$	$7.5_{4.7}$	$12.3_{12.6}$	$3.9_{0.6}$	$3.1_{1.0}$	$0.4_{0.0}$	$4.1_{1.8}$	$3.2_{0.7}$	$5.3_{0.3}$	$4.1_{0.5}$	$4.0_{0.3}$	$2.9_{0.7}$	$4.0_{0.3}$
Imbalanced	CLD	$0.9_{0.1}$	$2.0_{0.2}$	$3.6_{0.2}$	$2.7_{0.2}$	$7.8_{7.6}$	$6.0_{5.8}$	$4.9_{2.1}$	$4.1_{2.4}$	$0.4_{0.0}$	$4.0_{1.3}$	$3.1_{0.6}$	$4.9_{0.4}$	$5.1_{0.7}$	$3.4_{0.3}$	$2.4_{0.3}$	$3.7_{0.3}$
Imbalanced	DHC	$4.7_{3.6}$	$4.9_{1.8}$	$7.1_{3.1}$	$7.3_{2.8}$	$11.7_{1.6}$	$18.9_{1.5}$	$17.3_{5.9}$	$25.2_{3.1}$	$12.8_{16.5}$	$35.8_{31.1}$	$5.2_{1.3}$	$8.0_{2.5}$	$7.8_{2.2}$	$6.1_{1.5}$	$3.4_{0.6}$	$5.3_{1.8}$
Imbalanced	FCC	$1.3_{0.2}$	$1.8_{0.2}$	$3.6_{0.2}$	$3.0_{0.5}$	$6.9_{4.8}$	$2.9_{0.7}$	$7.2_{5.2}$	$3.1_{0.5}$	$0.4_{0.1}$	$3.9_{1.9}$	$3.6_{0.7}$	$4.7_{0.0}$	$3.3_{0.3}$	$4.1_{0.3}$	$2.4_{0.3}$	$3.4_{0.4}$
Imbalanced	GA-loss	$1.0_{0.1}$	$2.0_{0.0}$	$3.8_{0.0}$	$2.8_{0.1}$	$6.4_{2.6}$	$5.0_{1.6}$	$11.3_{1.1}$	$6.7_{2.6}$	$0.6_{0.0}$	$5.8_{0.8}$	$4.2_{0.3}$	$5.9_{0.3}$	$2.8_{0.4}$	$3.6_{0.3}$	$2.6_{0.2}$	$4.2_{0.3}$
Imbalanced	SKCDF	$0.7_{0.0}$	$2.1_{0.2}$	$3.9_{0.2}$	$3.7_{1.4}$	$14.6_{2.3}$	$11.0_{11.3}$	$10.0_{6.5}$	$3.8_{1.3}$	$3.9_{4.9}$	$8.0_{0.7}$	$4.0_{0.6}$	$5.7_{0.1}$	$10.3_{0.6}$	$5.1_{0.9}$	$2.9_{0.5}$	$3.6_{0.3}$
Imbalanced	Ours	$0.7_{0.1}$	$1.7_{0.1}$	$3.3_{0.1}$	$3.5_{0.4}$	$5.2_{1.2}$	$2.9_{0.6}$	$12.1_{11.7}$	$3.0_{0.6}$	$0.4_{0.0}$	$4.3_{0.7}$	$3.5_{0.4}$	$5.0_{0.6}$	$4.3_{0.9}$	$3.6_{0.1}$	$2.0_{0.2}$	$3.3_{0.4}$

Notes: Results are presented as mean_{std} over three independent runs. Full organ abbreviations are defined in the Supplemental Methods. The 'Ratio' row indicates the class imbalance ratio relative to the voxel count of the smallest class (Ad). V-Net serves as the fully supervised (100% labels) performance upper bound.

Table S6: Detailed per-class Mean Average Surface Distance (ASD, mm) on the FLARE22 dataset (zero-shot test), related to Table 1.

Organs		Li	St	Sp	LK	RK	Ao	IVC	Pa	Du	Ga	Es	LAG	RAG
Imbalance ratio		389.5	76.1	59.6	58.7	54.1	21.3	20.3	20.1	17.3	5.2	3.6	1.8	1.0
V-Net (100%)		$1.0_{0.1}$	$1.7_{0.5}$	$0.9_{0.3}$	$2.2_{0.5}$	$1.8_{0.8}$	$1.9_{0.1}$	$0.9_{0.1}$	$1.2_{0.1}$	$3.1_{0.1}$	$1.1_{0.7}$	$1.9_{0.1}$	$1.4_{0.1}$	$0.9_{0.1}$
General	UA-MT	$3.8_{0.7}$	$9.9_{1.2}$	$11.4_{3.7}$	$11.2_{2.8}$	$9.8_{0.5}$	$6.3_{2.5}$	$3.8_{1.5}$	$9.6_{1.5}$	$15.3_{1.1}$	$11.8_{1.6}$	$4.6_{0.5}$	$9.7_{3.8}$	$7.1_{0.7}$
General	CPS	$3.8_{0.4}$	$10.1_{0.9}$	$7.4_{1.5}$	$9.8_{0.7}$	$11.3_{1.3}$	$2.0_{0.8}$	$2.7_{0.1}$	$8.1_{1.2}$	$14.6_{1.4}$	$9.8_{1.3}$	$3.3_{0.8}$	$5.6_{0.8}$	$3.6_{1.0}$
General	DST	$2.7_{0.2}$	$15.2_{3.9}$	$8.7_{1.3}$	$18.5_{1.8}$	$13.7_{3.9}$	$2.1_{0.2}$	$8.3_{6.0}$	$18.2_{1.0}$	$19.9_{4.4}$	$17.8_{3.4}$	$5.5_{0.8}$	$69.1_{44.8}$	$3.8_{3.0}$
General	DMD	$3.6_{0.3}$	$9.0_{0.7}$	$5.6_{1.2}$	$8.9_{0.9}$	$12.5_{1.9}$	$1.5_{0.1}$	$3.1_{0.5}$	$10.8_{0.1}$	$14.3_{1.6}$	$11.9_{0.3}$	$3.0_{0.4}$	$4.8_{0.3}$	$3.0_{0.6}$
General	SLCNet	$2.5_{0.3}$	$12.1_{3.5}$	$11.8_{2.1}$	$10.3_{0.7}$	$9.6_{3.1}$	$5.7_{1.6}$	$3.5_{1.2}$	$8.1_{0.8}$	$14.0_{2.3}$	$15.2_{1.8}$	$6.3_{3.4}$	$8.1_{2.9}$	$3.7_{1.4}$
General	DyCON	$3.5_{0.4}$	$13.4_{1.5}$	$12.6_{3.3}$	$13.4_{1.7}$	$13.4_{1.9}$	$12.6_{3.1}$	$6.4_{1.9}$	$13.0_{6.4}$	$18.1_{4.0}$	$13.5_{2.8}$	$10.8_{2.5}$	$4.2_{5.9}$	$8.1_{1.0}$
Imbalanced	Adsh	$3.2_{0.5}$	$10.4_{0.4}$	$7.4_{1.4}$	$9.7_{1.5}$	$9.1_{1.1}$	$1.9_{0.1}$	$2.7_{0.5}$	$9.2_{0.6}$	$13.9_{1.0}$	$12.8_{3.8}$	$3.0_{0.4}$	$5.1_{0.2}$	$3.4_{0.5}$
Imbalanced	CLD	$3.4_{0.2}$	$8.5_{0.8}$	$8.1_{2.4}$	$8.0_{1.0}$	$11.5_{3.4}$	$1.7_{0.4}$	$2.2_{0.2}$	$8.0_{0.6}$	$12.2_{0.6}$	$12.4_{3.4}$	$2.8_{0.0}$	$5.3_{0.2}$	$3.4_{0.4}$
Imbalanced	DHC	$9.2_{0.4}$	$11.9_{2.1}$	$14.9_{2.4}$	$12.9_{1.1}$	$10.5_{1.7}$	$6.9_{3.6}$	$4.3_{1.4}$	$9.3_{1.6}$	$15.0_{3.8}$	$11.9_{1.7}$	$6.8_{4.5}$	$10.6_{0.9}$	$3.5_{0.9}$
Imbalanced	FCC	$4.6_{0.5}$	$9.5_{1.3}$	$8.2_{3.6}$	$8.4_{2.1}$	$13.3_{4.0}$	$1.8_{0.3}$	$3.5_{0.3}$	$7.6_{0.6}$	$13.1_{0.2}$	$10.6_{0.8}$	$2.6_{0.2}$	$5.8_{1.2}$	$3.1_{0.5}$
Imbalanced	GA-loss	$3.5_{0.4}$	$9.0_{1.3}$	$7.4_{2.4}$	$8.3_{0.8}$	$10.4_{1.2}$	$2.0_{0.2}$	$2.2_{0.2}$	$7.0_{0.9}$	$11.2_{1.2}$	$8.0_{1.2}$	$3.4_{0.5}$	$4.1_{0.6}$	$2.9_{0.5}$
Imbalanced	SKCDF	$3.3_{0.1}$	$13.2_{0.4}$	$12.3_{2.3}$	$15.2_{2.5}$	$11.8_{3.7}$	$4.1_{0.8}$	$4.0_{0.7}$	$12.7_{2.7}$	$16.1_{2.0}$	$20.0_{0.4}$	$4.4_{1.3}$	$11.8_{3.7}$	$7.6_{1.6}$
Imbalanced	Ours	$4.2_{0.3}$	$6.5_{0.7}$	$6.0_{0.7}$	$7.4_{0.6}$	$8.8_{2.2}$	$1.4_{0.2}$	$1.9_{0.2}$	$6.0_{0.9}$	$9.6_{0.2}$	$8.3_{1.0}$	$3.2_{0.2}$	$3.1_{0.3}$	$2.3_{0.4}$

Notes: Results are presented as mean_{std} over three independent runs. Full organ abbreviations are defined in the Supplemental Methods section. The imbalance ratio is relative to the voxel count of the smallest class (RAG).

Table S7: Experimental results on the WORD dataset under the 10% and 20% labeled data settings. Performance is evaluated using Dice (%) and ASD (mm). Related to Table 2.

Methods		WORD 10% Labeled				WORD 20% Labeled			
		Dice	ASD	$\text{Dice}_{\text{tail}}$	ASD_{tail}	Dice	ASD	$\text{Dice}_{\text{tail}}$	ASD_{tail}
V-Net (100%)		$84.19_{0.10}$	$0.89_{0.08}$	$71.75_{0.08}$	$1.40_{0.11}$	$84.19_{0.10}$	$0.89_{0.08}$	$71.75_{0.08}$	$1.40_{0.11}$
General	UA-MT	$60.82_{1.69}^{***}$	$7.01_{0.75}^{***}$	$38.74_{3.54}^{***}$	$9.83_{1.62}^{***}$	$62.39_{0.40}^{***}$	$7.39_{0.67}^{***}$	$41.61_{3.10}^{***}$	$8.50_{1.85}^{***}$
General	CPS	$77.28_{0.05}^{***}$	$1.94_{0.10}$	$60.80_{0.23}^{***}$	$2.28_{0.08}$	$80.85_{0.43}$	$1.57_{0.11}$	$65.80_{0.81}^{**}$	$1.99_{0.10}^{*}$
General	DST	$73.82_{1.20}^{***}$	$5.57_{2.20}^{***}$	$55.56_{3.23}^{***}$	$9.02_{7.75}^{***}$	$77.34_{2.20}^{***}$	$3.08_{1.08}^{***}$	$57.98_{6.58}^{***}$	$6.71_{4.27}^{***}$
General	DMD	$77.40_{0.85}^{**}$	$1.93_{0.13}$	$61.11_{1.52}^{***}$	$2.44_{0.40}$	$80.60_{0.36}^{**}$	$1.62_{0.14}$	$65.49_{0.40}^{***}$	$1.99_{0.02}^{**}$
General	SLCNet	$76.05_{1.05}^{***}$	$2.59_{0.36}^{***}$	$59.25_{1.49}^{***}$	$3.27_{0.24}^{***}$	$78.91_{1.23}^{***}$	$2.19_{0.40}^{***}$	$63.71_{1.96}^{***}$	$2.17_{0.18}^{***}$
General	DyCON	$53.50_{3.21}^{***}$	$7.98_{1.55}^{***}$	$24.15_{8.28}^{***}$	$12.05_{3.74}^{***}$	$49.93_{2.27}^{***}$	$7.25_{0.73}^{***}$	$14.32_{5.29}^{***}$	$8.92_{2.69}^{***}$
Imbalanced	Adsh	$77.12_{0.44}^{***}$	$2.02_{0.12}$	$60.26_{0.52}^{***}$	$2.40_{0.08}$	$79.83_{0.34}^{***}$	$1.74_{0.16}^{*}$	$64.63_{0.40}^{***}$	$1.99_{0.11}^{**}$
Imbalanced	CLD	$77.59_{0.85}^{**}$	$1.95_{0.14}$	$61.24_{0.92}^{***}$	$2.40_{0.21}$	$80.55_{0.21}^{**}$	$1.67_{0.08}$	$65.68_{0.29}^{***}$	$1.96_{0.03}^{**}$
Imbalanced	DHC	$72.16_{2.12}^{***}$	$4.78_{1.31}^{***}$	$58.01_{1.76}^{***}$	$4.39_{1.45}^{***}$	$75.69_{0.63}^{***}$	$3.51_{0.04}^{***}$	$61.70_{1.02}^{***}$	$2.76_{0.31}^{***}$
Imbalanced	FCC	$76.27_{0.87}^{***}$	$2.14_{0.18}^{**}$	$59.79_{1.36}^{***}$	$2.50_{0.19}^{**}$	$80.86_{0.08}$	$1.51_{0.06}$	$66.15_{0.32}^{**}$	$1.95_{0.07}^{*}$
Imbalanced	GA-loss	$73.85_{0.89}^{***}$	$2.69_{0.20}^{***}$	$57.43_{1.30}^{***}$	$2.69_{0.28}^{***}$	$72.47_{1.54}^{***}$	$4.35_{0.55}^{***}$	$61.71_{1.05}^{***}$	$2.12_{0.10}^{***}$
Imbalanced	SKCDF	$76.46_{1.01}^{***}$	$2.66_{0.18}^{***}$	$59.81_{2.04}^{***}$	$3.61_{0.35}^{***}$	$79.36_{0.51}^{***}$	$1.99_{0.17}^{***}$	$64.18_{0.95}^{***}$	$2.33_{0.18}^{***}$
Imbalanced	Ours	$77.96_{0.11}$	$1.98_{0.09}$	$62.82_{0.24}$	$2.29_{0.10}$	$81.00_{0.23}$	$1.62_{0.14}$	$66.90_{0.42}$	$1.83_{0.06}$

Notes: Results are presented as mean_{std} over three independent runs. Asterisks denote statistical significance from a paired Wilcoxon signed-rank test against our method ($^{*}p < 0.05,^{**}p < 0.01,^{***}p < 0.001$) after Benjamini-Hochberg correction. V-Net (100%) serves as the fully supervised performance upper bound.

Table S8: Generalization results on the FLARE22 dataset. Performance is evaluated using Dice (%) and ASD (mm). Related to Table 1.

Methods		FLARE22 (Directly test)			
		Dice	ASD	$\text{Dice}_{\text{tail}}$	ASD_{tail}
V-Net (100%)		$79.05_{0.65}$	$1.57_{0.10}$	$67.76_{1.03}$	$1.74_{0.13}$
General	UA-MT	$52.95_{0.39}^{***}$	$8.87_{0.54}^{***}$	$35.62_{1.15}^{***}$	$9.88_{0.32}^{***}$
	CPS	$48.87_{1.82}^{***}$	$7.31_{0.42}^{***}$	$32.20_{1.17}^{***}$	$7.91_{0.94}^{***}$
	DST	$37.87_{0.96}^{***}$	$14.20_{2.95}^{***}$	$20.67_{1.99}^{***}$	$21.33_{11.01}^{***}$
	DMD	$49.85_{1.16}^{***}$	$7.29_{0.35}^{***}$	$35.51_{0.70}^{***}$	$7.70_{0.48}^{***}$
	SLCNet	$54.47_{1.00}^{**}$	$8.60_{0.85}^{***}$	$36.66_{2.39}^{***}$	$9.67_{0.34}^{***}$
	DyCON	$46.22_{1.98}^{***}$	$11.70_{1.74}^{***}$	$24.60_{2.06}^{***}$	$12.98_{2.13}^{***}$
Imbalanced	Adsh	$49.06_{2.48}^{***}$	$7.16_{0.35}^{***}$	$32.90_{2.87}^{***}$	$7.81_{0.82}^{***}$
	CLD	$51.96_{1.49}^{***}$	$6.81_{0.46}^{***}$	$36.19_{0.27}^{***}$	$7.47_{0.72}^{***}$
	DHC	$51.28_{1.89}^{***}$	$10.08_{0.94}^{***}$	$36.67_{2.59}^{***}$	$10.16_{2.38}^{***}$
	FCC	$48.96_{2.10}^{***}$	$7.31_{0.78}^{***}$	$32.50_{1.22}^{***}$	$7.28_{0.62}^{***}$
	GA-loss	$55.63_{2.17}$	$6.24_{0.25}^{***}$	$39.91_{3.44}^{***}$	$6.12_{0.27}^{***}$
	SKCDF	$49.11_{0.57}^{***}$	$10.45_{1.02}^{***}$	$32.99_{0.61}^{***}$	$11.97_{1.63}^{***}$
	Ours	$56.59_{1.38}$	$5.36_{0.28}$	$43.53_{0.83}$	$5.52_{0.17}$

Notes: Results are presented as mean_{std} over three independent runs. Asterisks denote statistical significance from a paired Wilcoxon signed-rank test against our method ($^{*}p < 0.05, ^{**}p < 0.01, ^{***}p < 0.001$) after Benjamini-Hochberg correction.

Table S9: Comprehensive Penalized ASD results on the AMOS dataset. Performance is evaluated using ASD^{pen} (mm) and $\text{ASD}^{\text{pen}}_{\text{tail}}$ (mm). Related to Figure 2.

Methods		AMOS 5% Labeled		AMOS 10% Labeled		AMOS 20% Labeled	
		ASD^{pen}	$\text{ASD}^{\text{pen}}_{\text{tail}}$	ASD^{pen}	$\text{ASD}^{\text{pen}}_{\text{tail}}$	ASD^{pen}	$\text{ASD}^{\text{pen}}_{\text{tail}}$
V-Net (100%)		$1.46_{0.15}$	$2.21_{0.17}$	$1.46_{0.15}$	$2.21_{0.17}$	$1.46_{0.15}$	$2.21_{0.17}$
General	UA-MT	$13.77_{2.28}^{***}$	$14.02_{1.64}^{***}$	$9.92_{1.75}^{***}$	$9.45_{2.26}^{***}$	$8.49_{1.30}^{***}$	$9.68_{1.53}^{***}$
	CPS	$10.27_{0.89}^{***}$	$14.31_{1.01}^{***}$	$3.94_{0.25}^{***}$	$5.18_{0.18}^{***}$	$2.89_{0.18}^{***}$	$4.39_{0.27}^{***}$
	DST	$23.48_{3.72}^{***}$	$36.74_{10.79}^{***}$	$6.78_{0.81}^{***}$	$11.84_{4.43}^{***}$	$7.11_{1.64}^{***}$	$11.36_{2.66}^{***}$
	DMD	$8.64_{0.35}^{***}$	$11.31_{0.50}^{***}$	$3.72_{0.26}^{**}$	$5.05_{0.20}^{***}$	$3.09_{0.22}^{***}$	$4.64_{0.29}^{***}$
	SLCNet	$13.88_{1.33}^{***}$	$11.73_{0.90}^{***}$	$4.18_{0.34}^{***}$	$5.07_{0.36}^{***}$	$4.63_{2.62}^{***}$	$4.03_{0.22}^{**}$
	DyCON	$23.35_{0.39}^{***}$	$33.25_{1.91}^{***}$	$11.60_{2.64}^{***}$	$13.27_{4.48}^{***}$	$24.68_{4.55}^{***}$	$52.69_{9.07}^{***}$
Imbalanced	Adsh	$10.75_{0.74}^{***}$	$15.09_{2.46}^{***}$	$3.64_{0.11}^{***}$	$5.17_{0.07}^{***}$	$3.18_{0.29}^{***}$	$4.49_{0.43}^{***}$
	CLD	$8.52_{0.24}^{***}$	$11.73_{1.23}^{***}$	$3.56_{0.13}$	$4.51_{0.70}^{***}$	$2.99_{0.16}^{***}$	$4.09_{0.09}^{***}$
	DHC	$11.22_{0.63}^{***}$	$11.88_{0.83}^{***}$	$7.81_{1.48}^{***}$	$5.19_{0.51}^{***}$	$5.72_{0.44}^{***}$	$4.54_{0.24}^{***}$
	FCC	$8.63_{0.53}^{***}$	$12.75_{1.46}^{***}$	$3.53_{0.17}^{*}$	$4.65_{0.09}^{***}$	$3.41_{0.21}^{***}$	$4.79_{0.23}^{***}$
	GA-loss	$8.92_{1.22}^{***}$	$10.67_{2.18}^{***}$	$3.42_{0.19}^{*}$	$5.04_{0.21}^{***}$	$3.45_{0.33}^{***}$	$4.36_{0.50}^{***}$
	SKCDF	$10.59_{0.62}^{***}$	$12.16_{0.32}^{***}$	$4.61_{0.27}^{***}$	$5.55_{0.45}^{***}$	$5.11_{0.30}^{***}$	$5.77_{0.62}^{***}$
	Ours	$6.43_{0.32}$	$7.65_{0.45}$	$3.29_{0.05}$	$4.16_{0.38}$	$2.53_{0.09}$	$4.02_{0.06}$

Notes: Results are presented as mean_{std} over three independent runs. Asterisks denote statistical significance from a paired Wilcoxon signed-rank test against our method ($^{*}p < 0.05, ^{**}p < 0.01, ^{***}p < 0.001$) after Benjamini-Hochberg correction.

Table S10: Comprehensive Penalized ASD results on the WORD dataset. Performance is evaluated using ASD^{pen} (mm) and $\text{ASD}^{\text{pen}}_{\text{tail}}$ (mm). Related to Figure 2.

Methods		WORD 5% Labeled		WORD 10% Labeled		WORD 20% Labeled	
		ASD^{pen}	$\text{ASD}^{\text{pen}}_{\text{tail}}$	ASD^{pen}	$\text{ASD}^{\text{pen}}_{\text{tail}}$	ASD^{pen}	$\text{ASD}^{\text{pen}}_{\text{tail}}$
V-Net (100%)		$0.89_{0.08}$	$1.40_{0.11}$	$0.89_{0.08}$	$1.40_{0.11}$	$0.89_{0.08}$	$1.40_{0.11}$
General	UA-MT	$16.66_{1.67}^{***}$	$21.24_{4.76}^{***}$	$7.27_{0.93}^{***}$	$10.67_{1.17}^{***}$	$8.47_{1.15}^{***}$	$11.99_{3.23}^{***}$
	CPS	$5.21_{0.34}^{**}$	$4.16_{0.39}$	$1.94_{0.10}$	$2.28_{0.08}$	$1.57_{0.11}$	$1.99_{0.10}^{*}$
	DST	$10.98_{3.23}^{***}$	$18.46_{8.80}^{***}$	$5.57_{2.20}^{***}$	$9.02_{7.75}^{***}$	$5.13_{3.93}^{***}$	$12.73_{12.67}^{***}$
	DMD	$5.23_{0.41}^{**}$	$4.01_{0.63}$	$1.93_{0.13}$	$2.44_{0.40}$	$1.62_{0.14}$	$1.99_{0.02}^{**}$
	SLCNet	$4.77_{0.46}^{*}$	$4.07_{0.42}$	$2.59_{0.36}^{***}$	$3.27_{0.24}^{***}$	$2.19_{0.40}^{***}$	$2.17_{0.18}^{***}$
	DyCON	$43.49_{8.30}^{***}$	$66.46_{18.33}^{***}$	$14.40_{3.92}^{***}$	$31.41_{14.73}^{***}$	$22.48_{4.26}^{***}$	$56.96_{12.32}^{***}$
Imbalanced	Adsh	$5.59_{0.48}^{***}$	$4.04_{0.23}^{*}$	$2.02_{0.12}$	$2.40_{0.08}$	$1.74_{0.16}^{*}$	$1.99_{0.11}^{**}$
	CLD	$4.96_{0.25}^{**}$	$3.90_{0.11}^{*}$	$1.95_{0.14}$	$2.40_{0.21}$	$1.67_{0.08}$	$1.96_{0.03}^{**}$
	DHC	$13.86_{7.08}^{***}$	$12.47_{9.62}^{***}$	$4.78_{1.31}^{***}$	$4.39_{1.45}^{***}$	$3.51_{0.04}^{***}$	$2.76_{0.31}^{***}$
	FCC	$4.61_{0.90}^{*}$	$3.81_{0.43}$	$2.21_{0.14}^{***}$	$2.50_{0.19}^{**}$	$1.51_{0.06}$	$1.95_{0.07}^{*}$
	GA-loss	$5.38_{0.17}^{***}$	$4.03_{0.27}$	$2.69_{0.20}^{***}$	$2.69_{0.28}^{***}$	$4.35_{0.55}^{***}$	$2.12_{0.10}^{***}$
	SKCDF	$6.43_{0.46}^{***}$	$5.75_{0.51}^{***}$	$2.66_{0.18}^{***}$	$3.61_{0.35}^{***}$	$1.99_{0.17}^{***}$	$2.33_{0.18}^{***}$
	Ours	$4.61_{0.57}$	$4.29_{0.15}$	$1.98_{0.09}$	$2.29_{0.10}$	$1.62_{0.14}$	$1.83_{0.06}$

Notes: Results are presented as mean_{std} over three independent runs. Asterisks denote statistical significance from a paired Wilcoxon signed-rank test against our method ($^{*}p < 0.05, ^{**}p < 0.01, ^{***}p < 0.001$) after Benjamini-Hochberg correction.

Table S11: Step-wise ablation study on the AMOS 5% labeled dataset. Performance is evaluated using Dice (%) and ASD (mm). Related to Figure 4.

Configuration	Dice	ASD	Dice$_{\text{tail}}$	ASD$_{\text{tail}}$
Baseline (CPS)	$57.98_{1.28}^{***}$	$5.52_{0.56}^{***}$	$41.16_{0.40}^{***}$	$5.24_{0.65}^{***}$
+ DAFR	$60.53_{1.56}^{***}$	$4.93_{0.47}^{***}$	$44.84_{0.34}^{***}$	$4.47_{0.52}^{***}$
+ RLM	$62.85_{0.52}^{***}$	$4.82_{0.26}^{***}$	$47.62_{0.90}^{***}$	$4.38_{0.36}^{**}$
Full Model (DuoMod-Net)	$65.22_{0.59}$	$4.40_{0.13}$	$51.37_{0.57}$	$3.39_{0.37}$

Notes: Results are presented as mean$_{std}$ over three independent runs. Asterisks denote statistical significance from a paired Wilcoxon signed-rank test against our method ($^{*}p < 0.05,^{**}p < 0.01,^{***}p < 0.001$) after Benjamini-Hochberg correction.

Table S12: Mechanism verification of the DAFR (a) and RLM (b) modules on the AMOS dataset under the 5% labeled setting. Performance is evaluated using Dice (%) and ASD (mm). Related to Figure 4.

Configuration / Variant	Dice	ASD	Dice$_{\text{tail}}$	ASD$_{\text{tail}}$
(a) DAFR Mechanism Analysis				
Baseline (CPS)	$57.98_{1.28}^{***}$	$5.52_{0.56}^{**}$	$41.16_{0.40}^{***}$	$5.24_{0.65}^{***}$
Gaussian Noise ($\sigma = 0.1$)	$61.32_{0.85}^{***}$	$4.68_{0.18}^{**}$	$46.75_{1.84}^{***}$	$3.89_{0.26}^{***}$
Gaussian Noise ($\sigma = 0.5$)	$62.26_{0.92}^{***}$	$4.88_{0.36}^{***}$	$46.63_{1.87}^{***}$	$3.76_{0.24}^{***}$
DAFR (Ours)	$65.22_{0.59}$	$4.40_{0.13}$	$51.37_{0.57}$	$3.39_{0.37}$
(b) RLM Weighting Strategy Analysis				
Baseline (CPS)	$57.98_{1.28}^{***}$	$5.52_{0.56}^{**}$	$41.16_{0.40}^{***}$	$5.24_{0.65}^{***}$
Linear Weighting	$62.74_{0.91}^{***}$	$4.82_{0.14}^{***}$	$46.77_{1.75}^{***}$	$4.47_{0.21}^{***}$
CReST Weighting	$61.54_{1.07}^{***}$	$4.53_{0.33}^{*}$	$45.44_{1.26}^{***}$	$4.42_{0.69}^{***}$
RLM (Ours)	$65.22_{0.59}$	$4.40_{0.13}$	$51.37_{0.57}$	$3.39_{0.37}$

Notes: Results are presented as mean$_{std}$ over three independent runs. Asterisks denote statistical significance from a paired Wilcoxon signed-rank test against our method ($^{*}p < 0.05,^{**}p < 0.01,^{***}p < 0.001$) after Benjamini-Hochberg correction.

Table S13: Hyperparameter sensitivity analysis of the DAFR strength (τ) on AMOS and WORD datasets under the 5% labeled setting. Performance is evaluated using Dice (%) and ASD (mm). Related to Figure 4.

Dataset	DAFR Strength (τ)	Dice	ASD	Dice$_{\text{tail}}$	ASD$_{\text{tail}}$
AMOS	$\tau = 0.1$	$61.66_{1.62}^{***}$	$4.74_{0.35}^{***}$	$48.13_{1.93}^{***}$	$3.62_{0.32}^{***}$
	$\tau = 0.5$	$63.51_{0.94}^{***}$	$4.70_{0.09}$	$48.90_{1.23}^{***}$	$3.90_{0.20}^{**}$
	$\tau = 1.0$ (Default)	$65.22_{0.59}$	$4.40_{0.13}$	$51.37_{0.57}$	$3.39_{0.37}$
	$\tau = 2.0$	$65.12_{0.64}$	$4.40_{0.14}^{***}$	$50.41_{1.56}$	$3.97_{0.37}^{***}$
WORD	$\tau = 0.1$	$70.91_{0.17}^{***}$	$3.54_{0.31}^{*}$	$54.72_{0.76}^{***}$	$4.07_{0.23}^{*}$
	$\tau = 0.5$	$71.82_{0.23}^{***}$	$4.12_{0.57}^{***}$	$56.12_{0.63}$	$4.27_{0.17}^{***}$
	$\tau = 1.0$ (Default)	$72.66_{0.25}$	$3.76_{0.72}$	$56.37_{0.37}$	$3.67_{0.15}$
	$\tau = 2.0$	$71.95_{0.34}^{***}$	$3.65_{0.42}^{*}$	$55.92_{0.72}$	$3.93_{0.18}^{**}$

Notes: Results are presented as mean$_{std}$ over three independent runs. Asterisks denote statistical significance from a paired Wilcoxon signed-rank test against the default setting ($^{*}p < 0.05,^{**}p < 0.01,^{***}p < 0.001$) after Benjamini-Hochberg correction.

Table S14: Sensitivity analysis of the RLM weight floor (ω_{floor}) on the AMOS dataset under the 5% labeled setting. Performance is evaluated using Dice (%) and ASD (mm). Related to Figure 4.

Weight Floor (ω_{floor})	Dice	ASD	Dice$_{\text{tail}}$	ASD$_{\text{tail}}$
0.01	$62.41_{0.83}^{***}$	$5.01_{0.29}^{***}$	$49.03_{0.91}^{***}$	$4.54_{0.33}^{***}$
0.05 (Default)	$65.22_{0.59}$	$4.40_{0.13}$	$51.37_{0.57}$	$3.39_{0.37}$
0.10	$65.06_{0.17}$	$4.40_{0.06}$	$49.93_{0.82}^{***}$	$3.84_{0.25}^{**}$

Notes: Results are presented as mean$_{std}$ over three independent runs. Asterisks denote statistical significance from a paired Wilcoxon signed-rank test against the default setting ($^{*}p < 0.05,^{**}p < 0.01,^{***}p < 0.001$) after Benjamini-Hochberg correction.

Table S15: Sensitivity analysis of intensity clipping percentiles on the AMOS dataset under the 5% labeled setting. Performance is evaluated using Dice (%) and ASD (mm). Related to Figure 4.

Configuration	Dice	ASD	Dice$_{\text{tail}}$	ASD$_{\text{tail}}$
Clip0_100	$64.61_{0.26}^{***}$	$4.39_{0.42}$	$49.71_{0.34}^{***}$	$4.08_{0.64}^{***}$
Clip5_95 (Default)	$65.22_{0.59}$	$4.40_{0.13}$	$51.37_{0.57}$	$3.39_{0.37}$
Clip10_90	$61.54_{0.85}^{***}$	$4.84_{0.19}^{***}$	$48.47_{0.78}^{***}$	$4.76_{0.51}^{***}$

Notes: Results are presented as mean$_{std}$ over three independent runs. Asterisks denote statistical significance from a paired Wilcoxon signed-rank test against the default setting ($^{*}p < 0.05,^{**}p < 0.01,^{***}p < 0.001$) after Benjamini-Hochberg correction.
